# Supplementary material for: Phosphorylation of seryl-tRNA synthetase by ATM/ATR is essential for hypoxia-induced angiogenesis
Source: PLoS Biol. 2020 Dec 22;18(12):e3000991. doi: 10.1371/journal.pbio.3000991 (PMC7755189; doi:10.1371/journal.pbio.3000991)
Supplement: S2 Data — (PPTX) [file pbio.3000991.s013.pptx]

## Slide 1
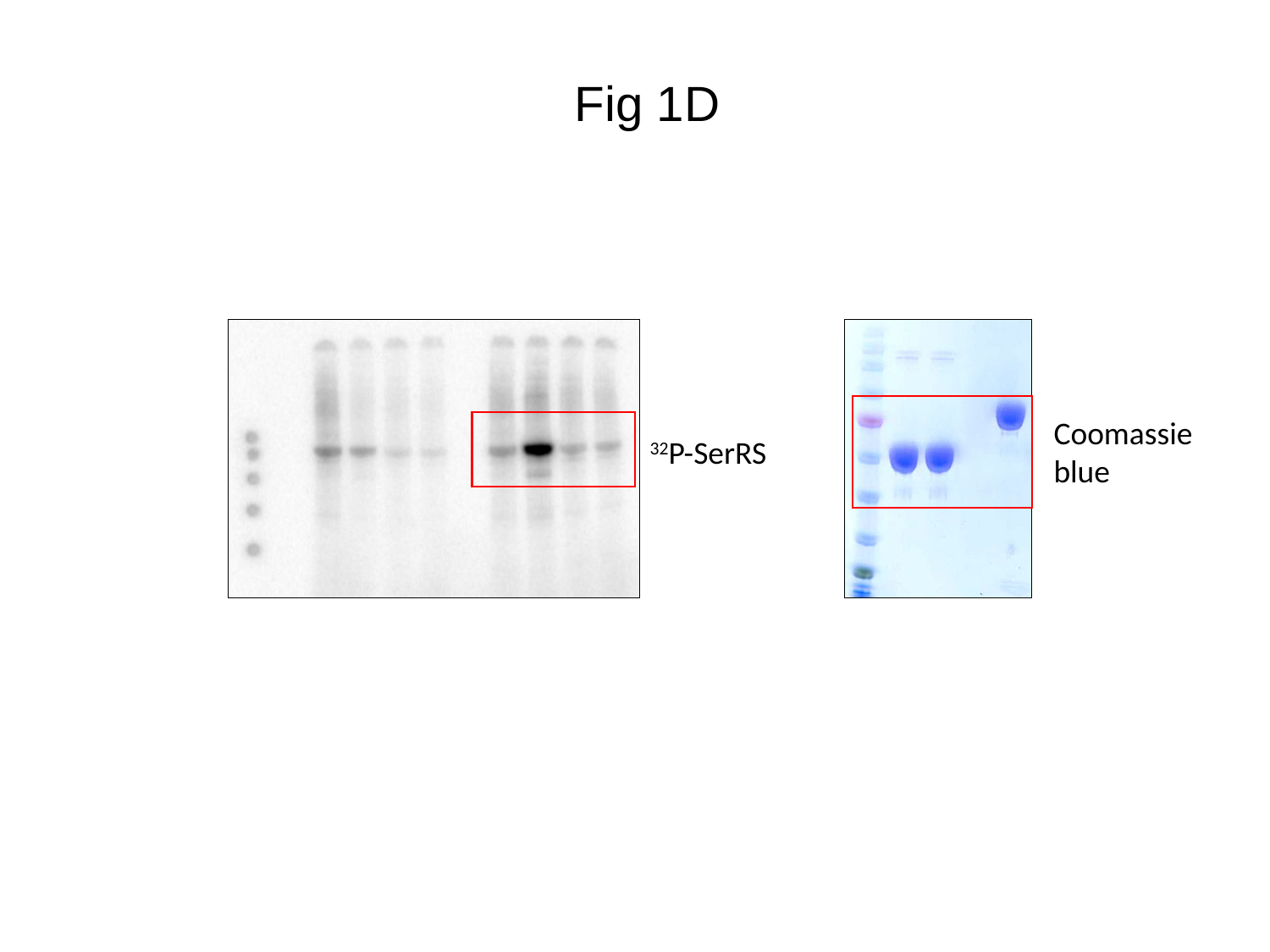

Fig 1D
Coomassie
blue
32P-SerRS

## Slide 2
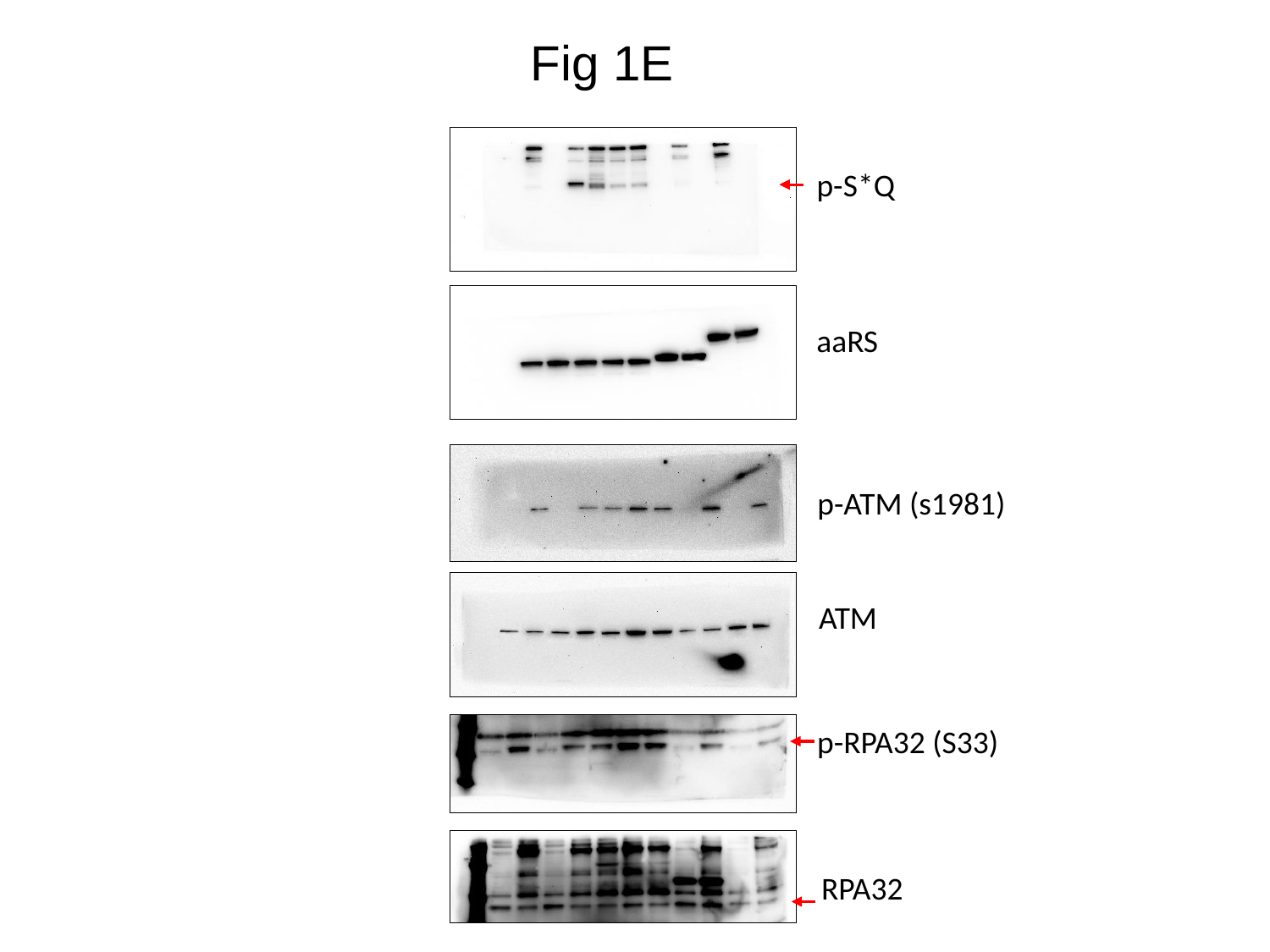

Fig 1E
p-S*Q
aaRS
p-ATM (s1981)
ATM
p-RPA32 (S33)
RPA32

## Slide 3
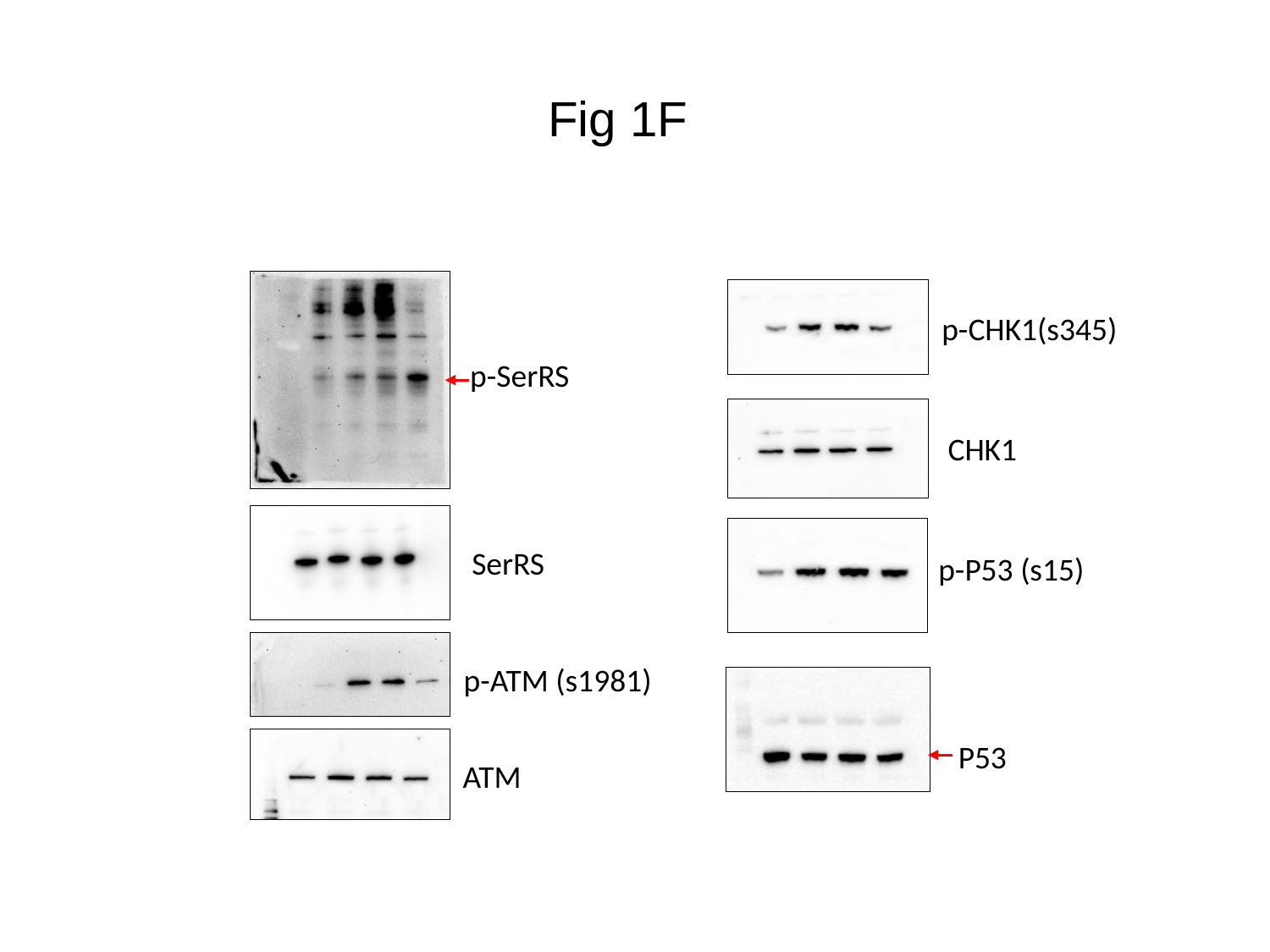

Fig 1F
p-CHK1(s345)
p-SerRS
CHK1
SerRS
p-P53 (s15)
p-ATM (s1981)
P53
ATM

## Slide 4
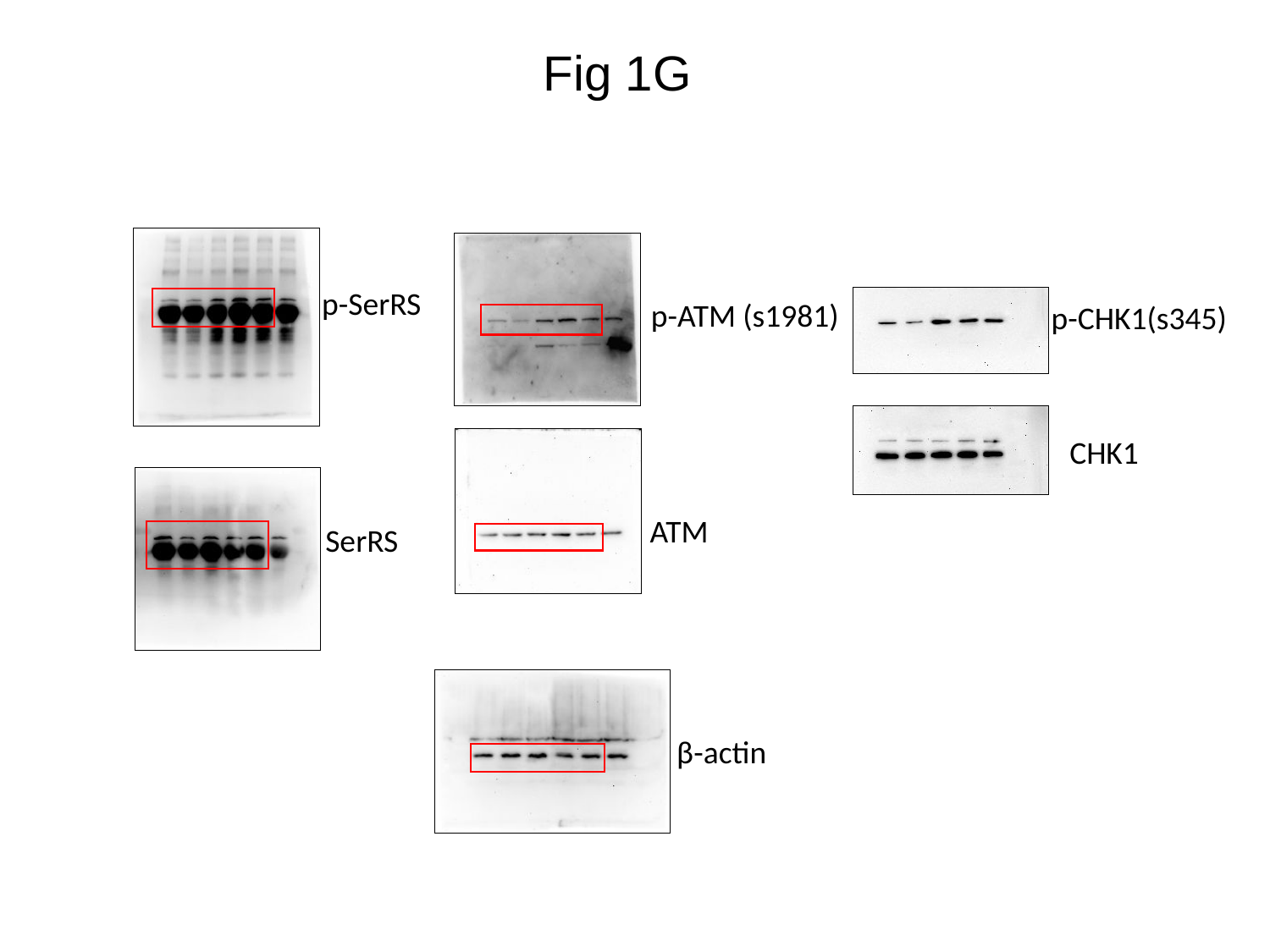

Fig 1G
p-SerRS
p-ATM (s1981)
p-CHK1(s345)
CHK1
ATM
SerRS
β-actin

## Slide 5
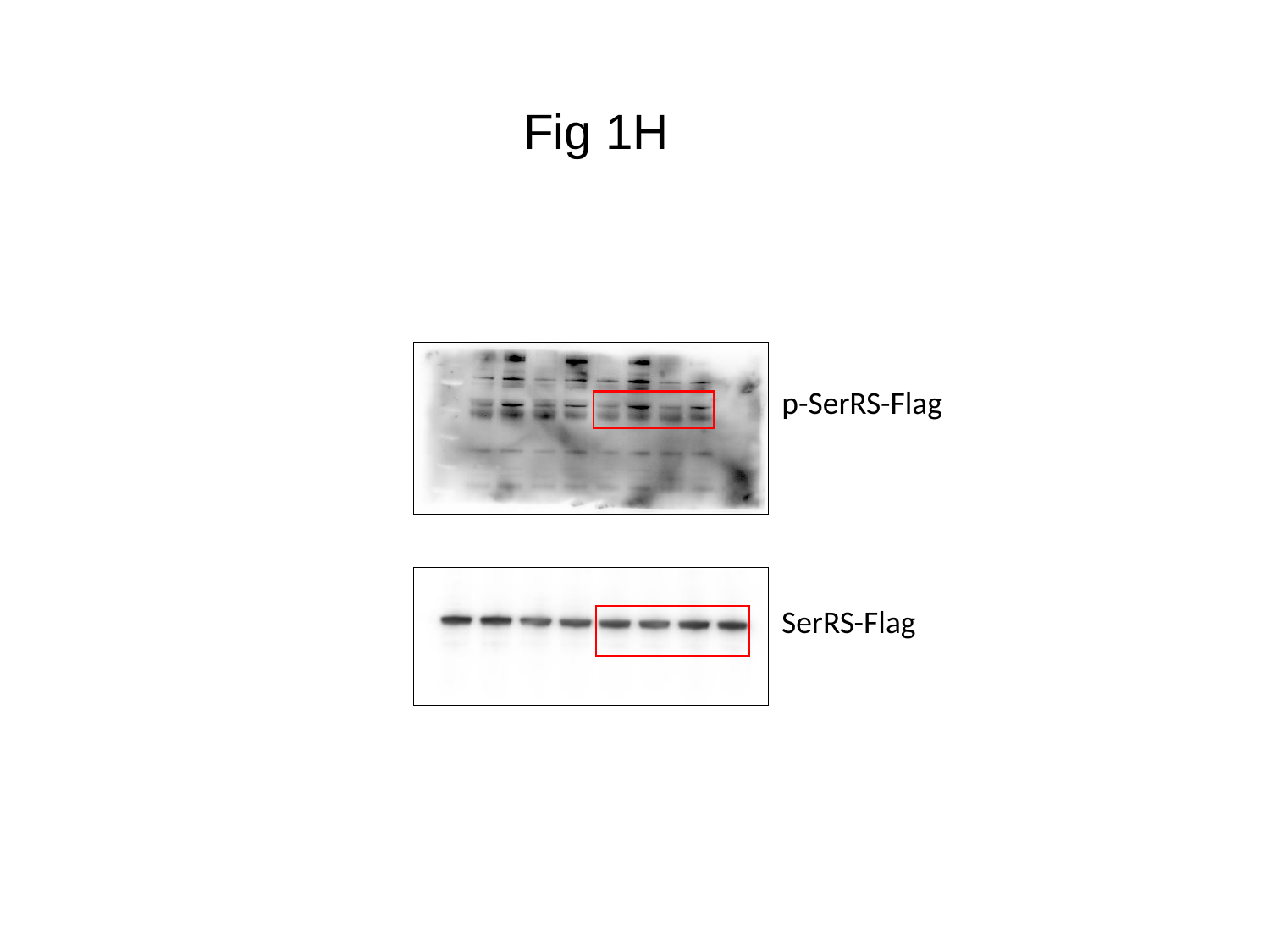

Fig 1H
p-SerRS-Flag
SerRS-Flag

## Slide 6
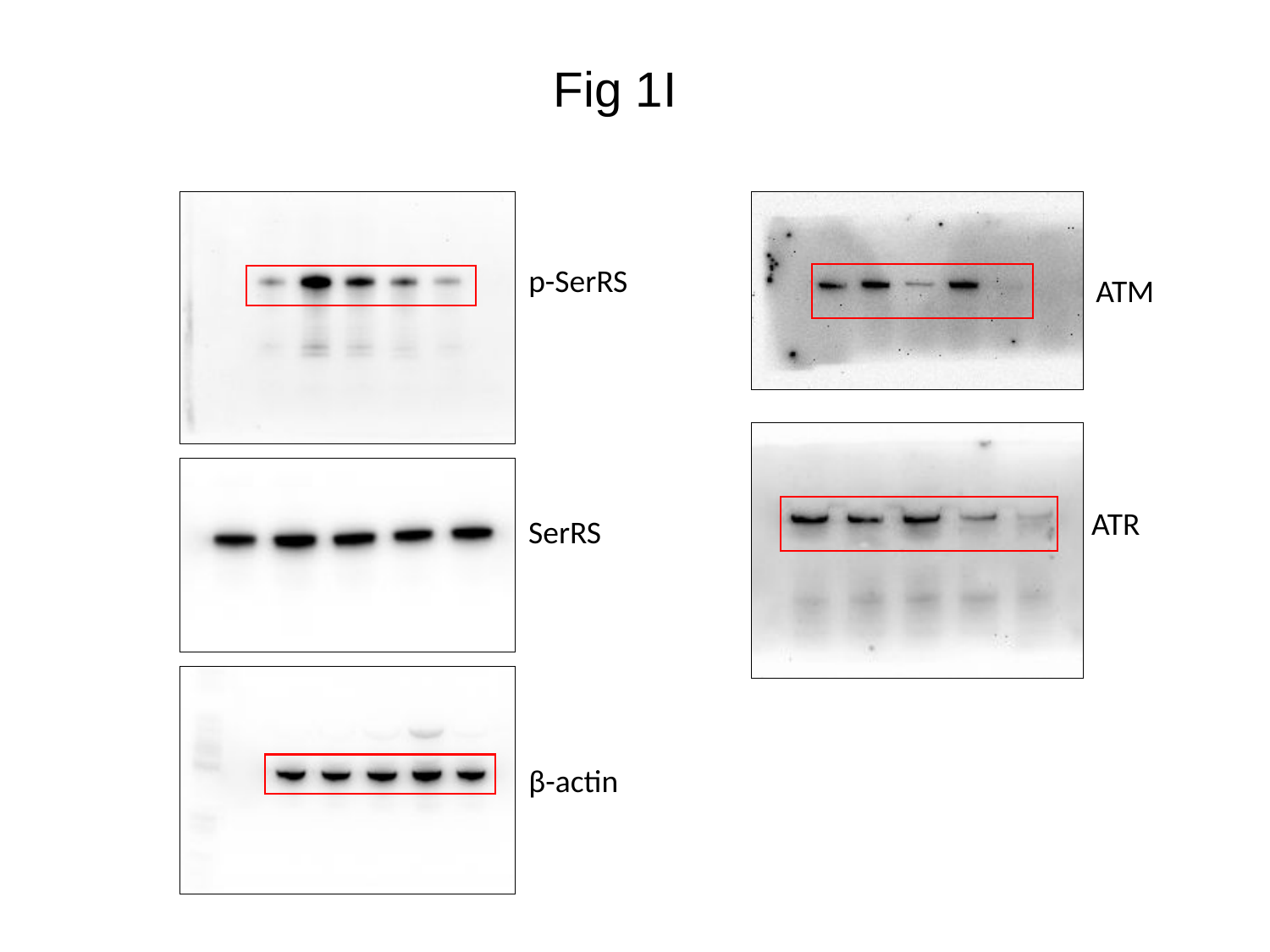

Fig 1I
p-SerRS
ATM
ATR
SerRS
β-actin

## Slide 7
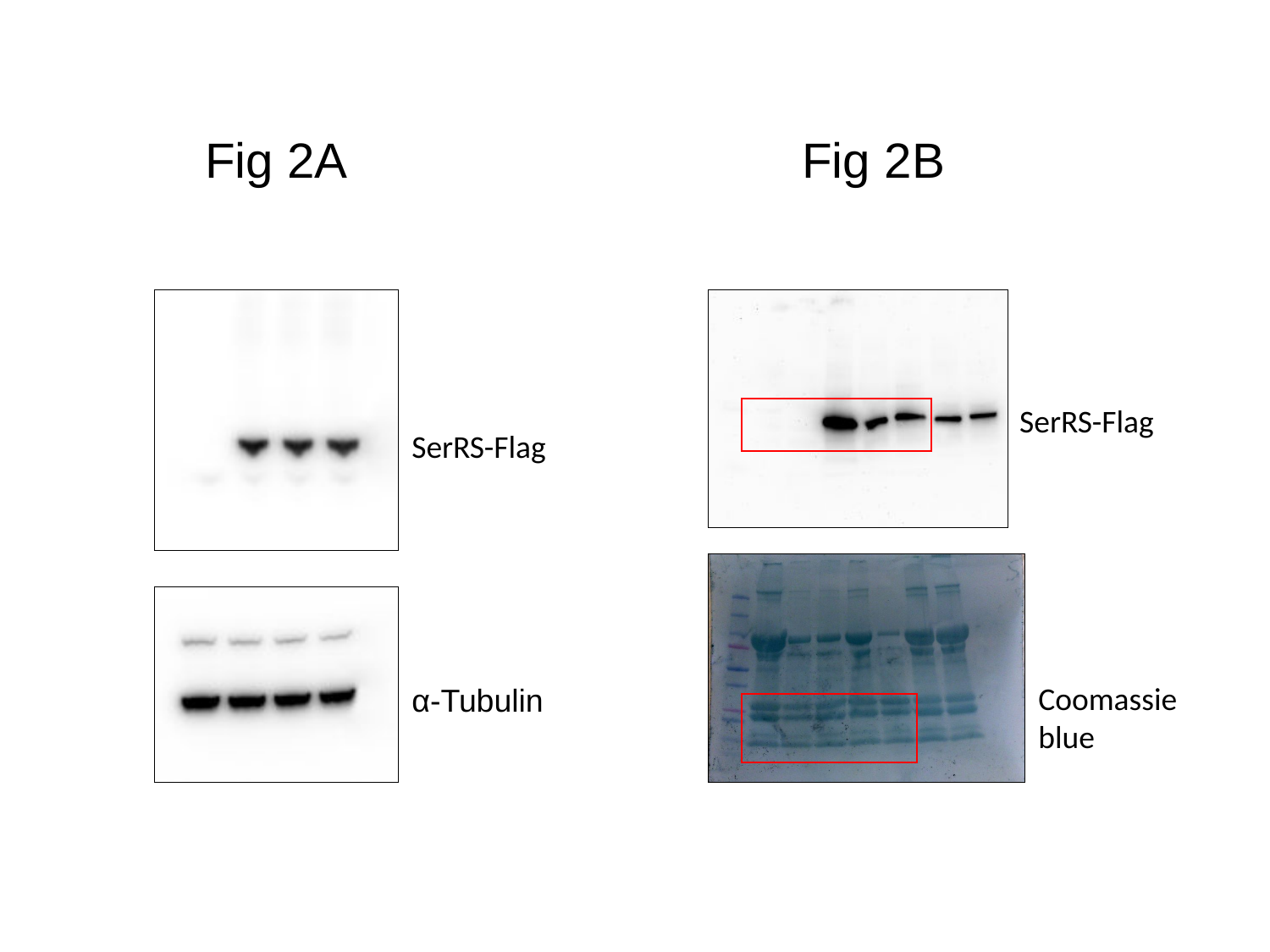

Fig 2A
Fig 2B
SerRS-Flag
SerRS-Flag
Coomassie
blue
α-Tubulin

## Slide 8
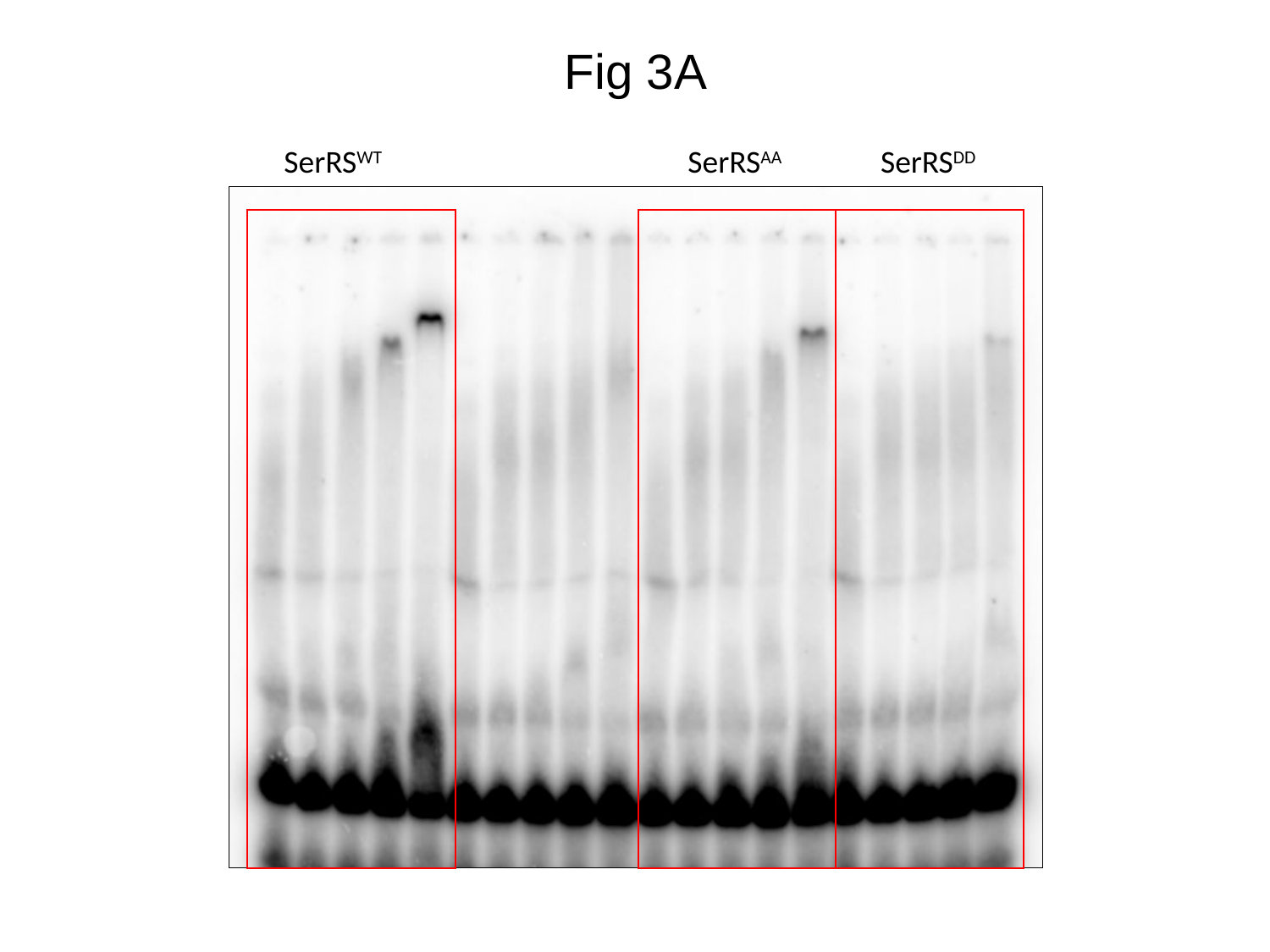

Fig 3A
SerRSWT
SerRSAA
SerRSDD

## Slide 9
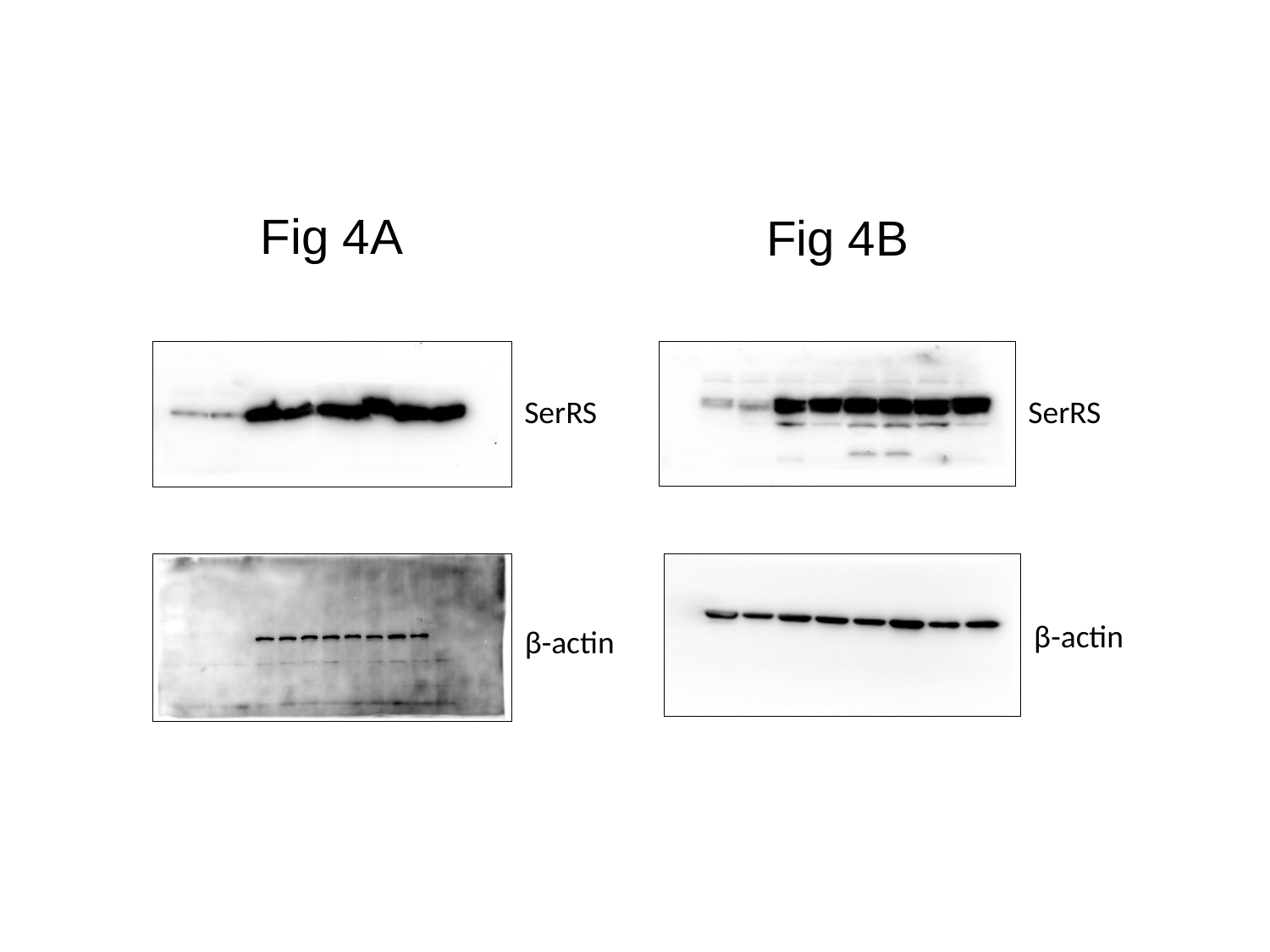

Fig 4A
Fig 4B
SerRS
SerRS
β-actin
β-actin

## Slide 10
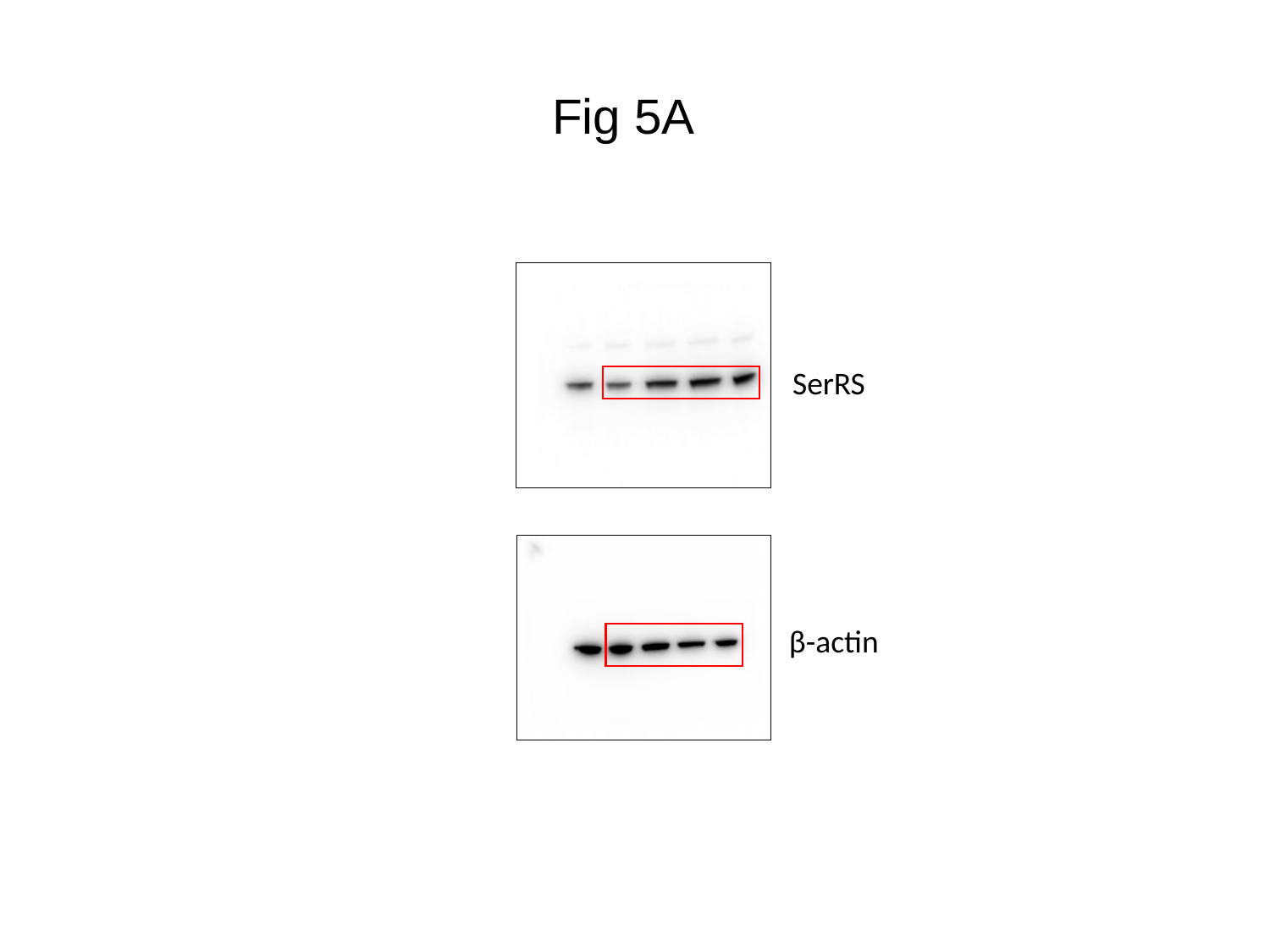

Fig 5A
SerRS
β-actin

## Slide 11
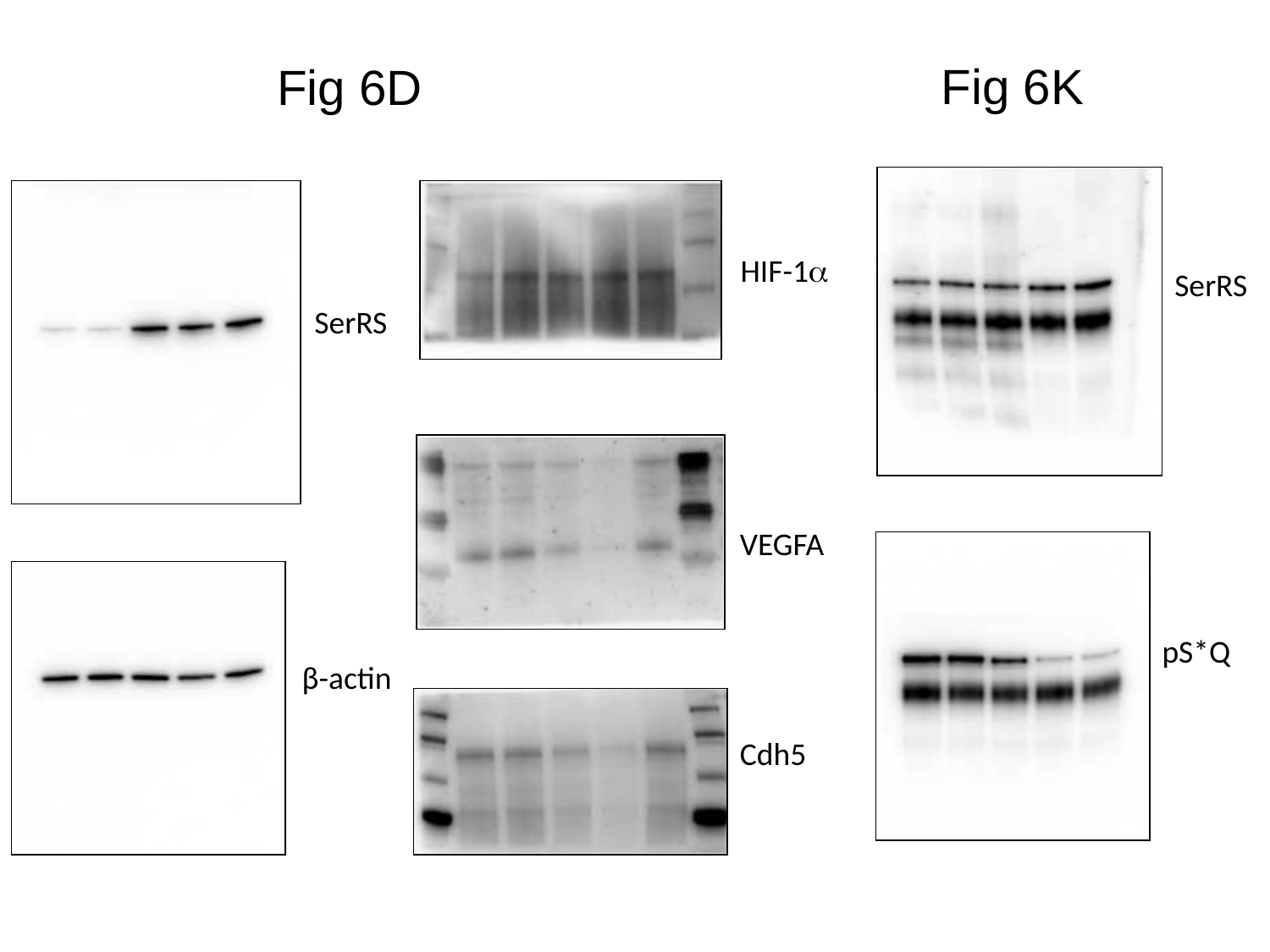

Fig 6K
Fig 6D
HIF-1a
SerRS
SerRS
VEGFA
pS*Q
β-actin
Cdh5

## Slide 12
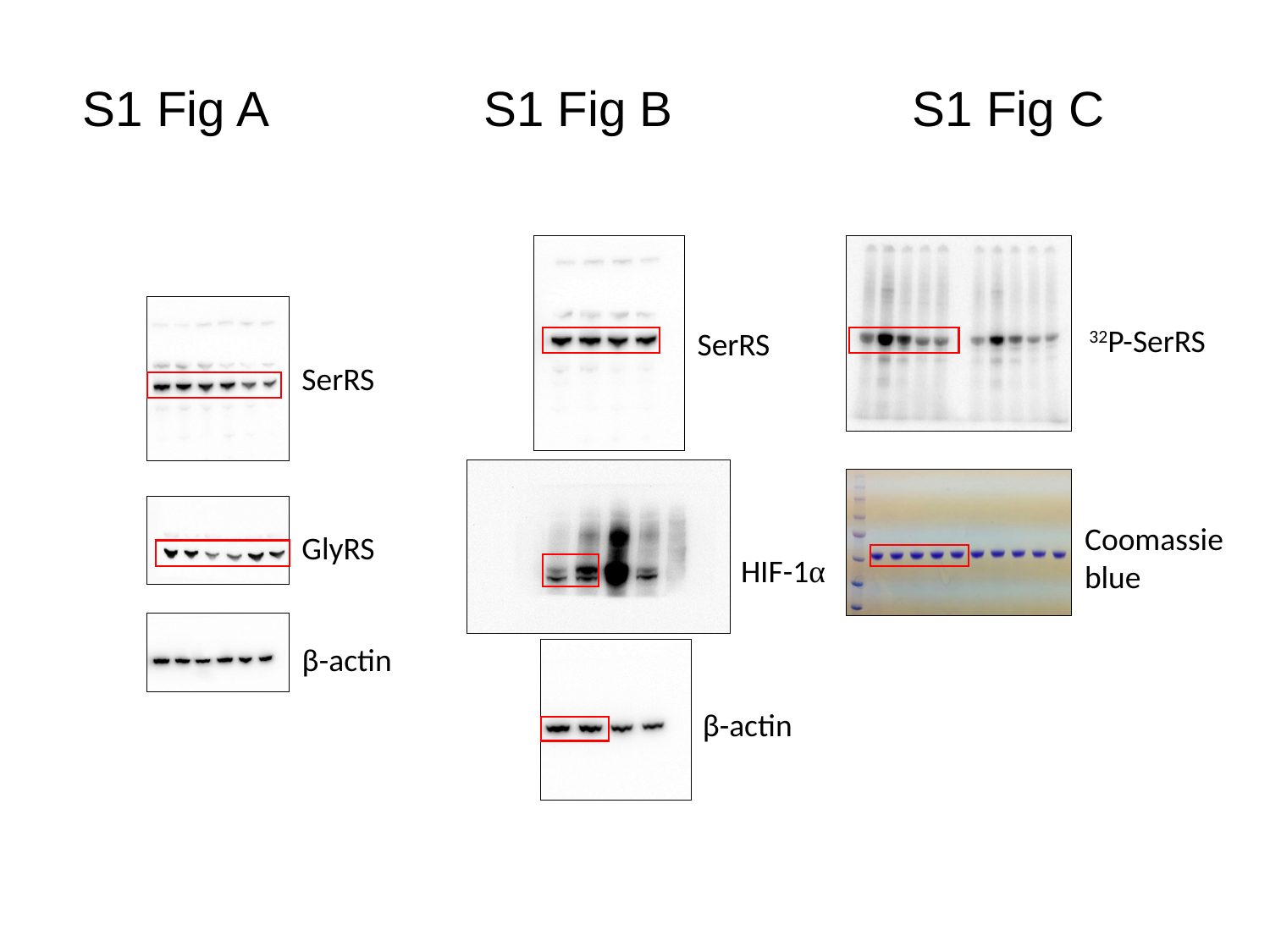

S1 Fig B
S1 Fig C
S1 Fig A
32P-SerRS
SerRS
SerRS
Coomassie
blue
GlyRS
HIF-1α
β-actin
β-actin

## Slide 13
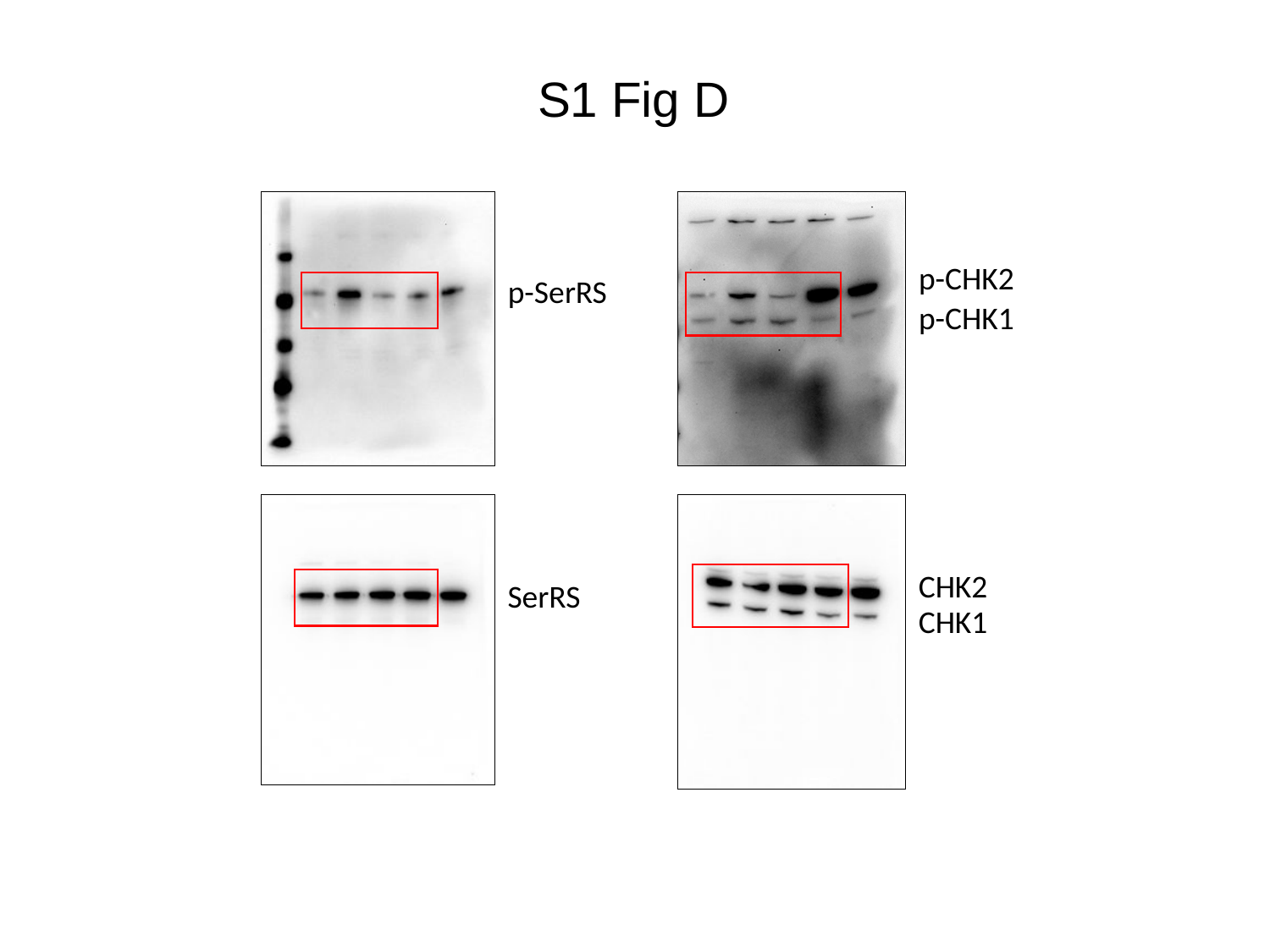

S1 Fig D
p-CHK2
p-SerRS
p-CHK1
CHK2
SerRS
CHK1

## Slide 14
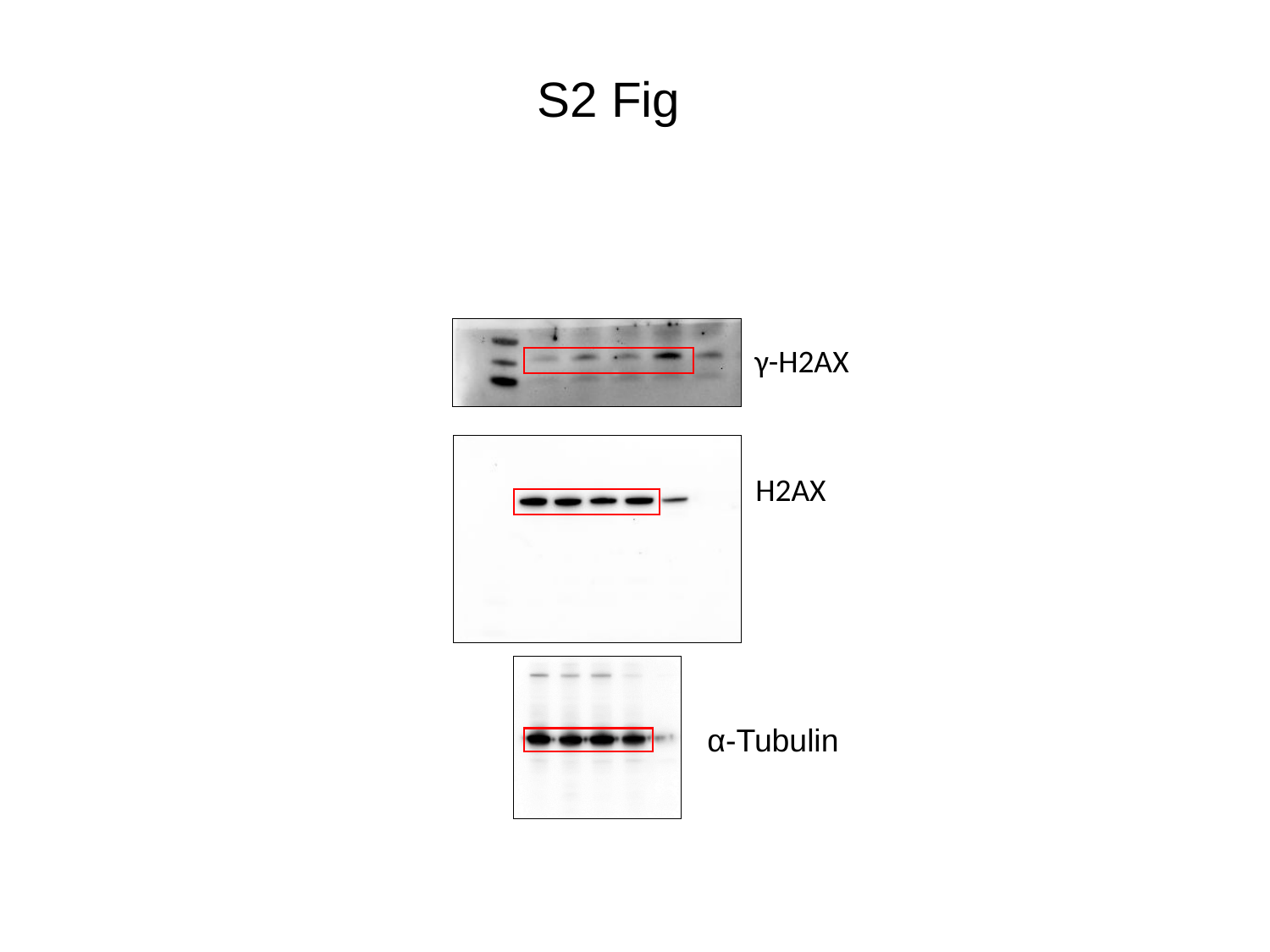

S2 Fig
γ-H2AX
H2AX
α-Tubulin

## Slide 15
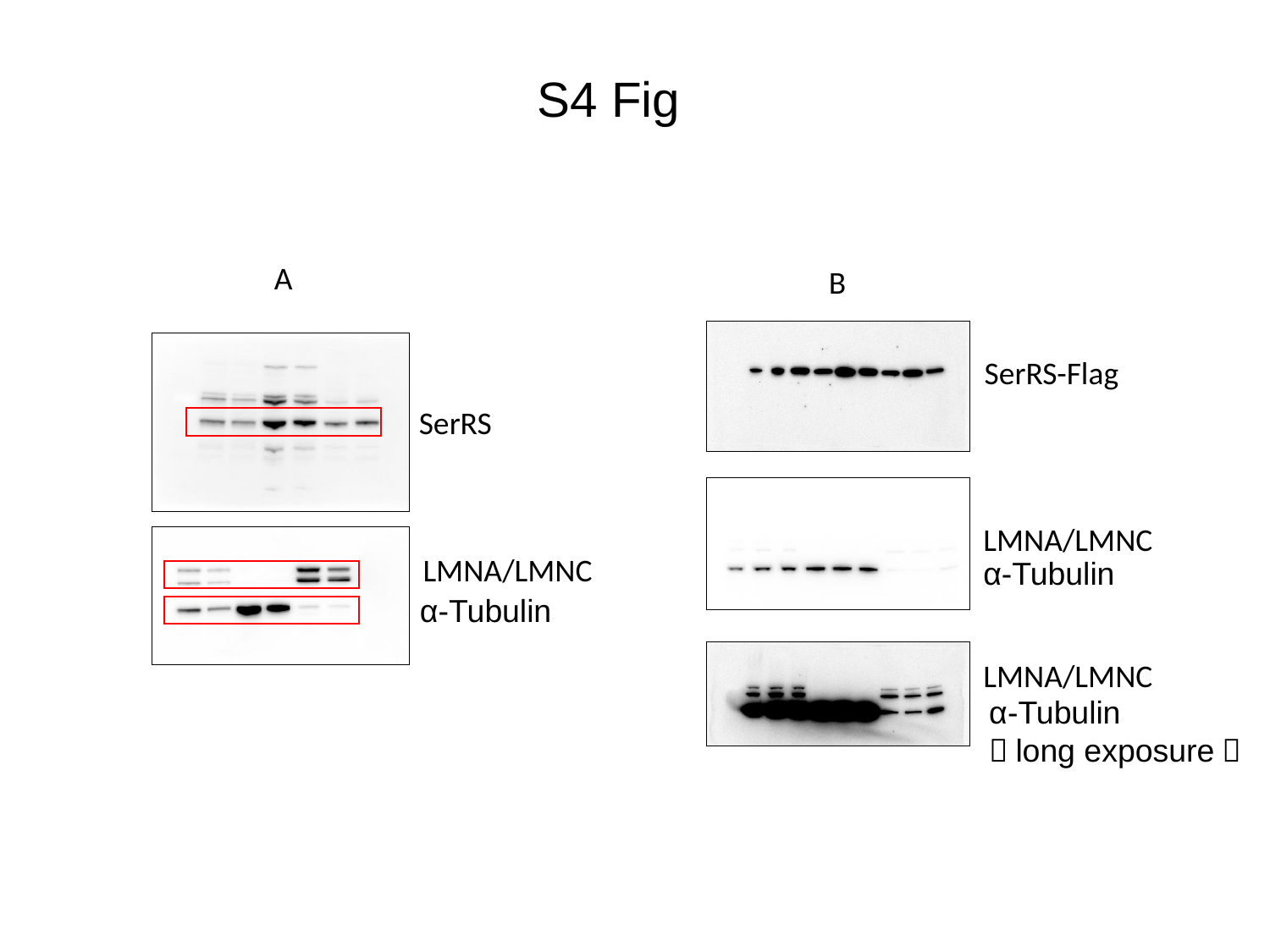

S4 Fig
A
B
SerRS-Flag
SerRS
LMNA/LMNC
LMNA/LMNC
α-Tubulin
α-Tubulin
LMNA/LMNC
α-Tubulin
（long exposure）

## Slide 16
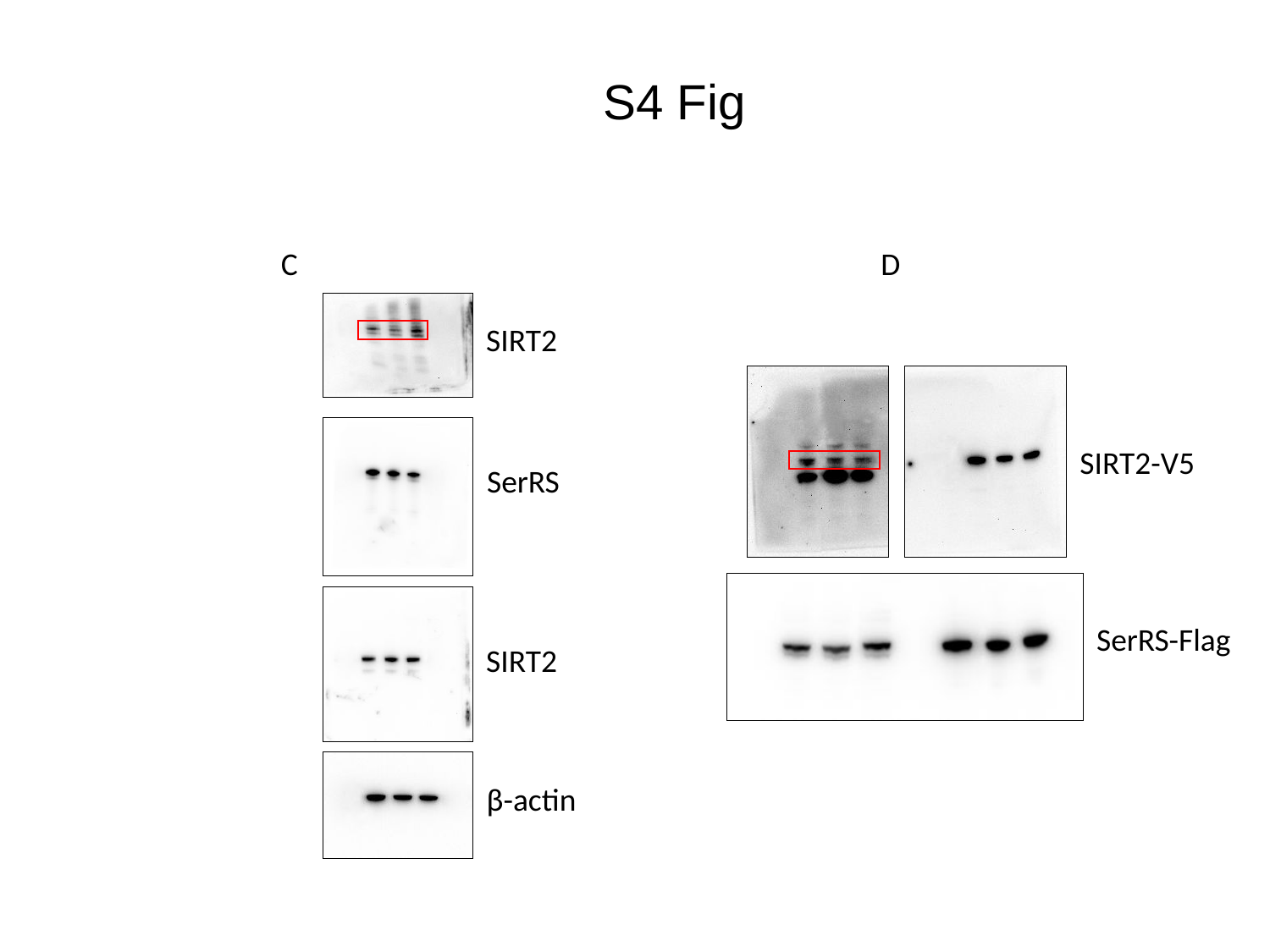

S4 Fig
C
D
SIRT2
SIRT2-V5
SerRS
SerRS-Flag
SIRT2
β-actin

## Slide 17
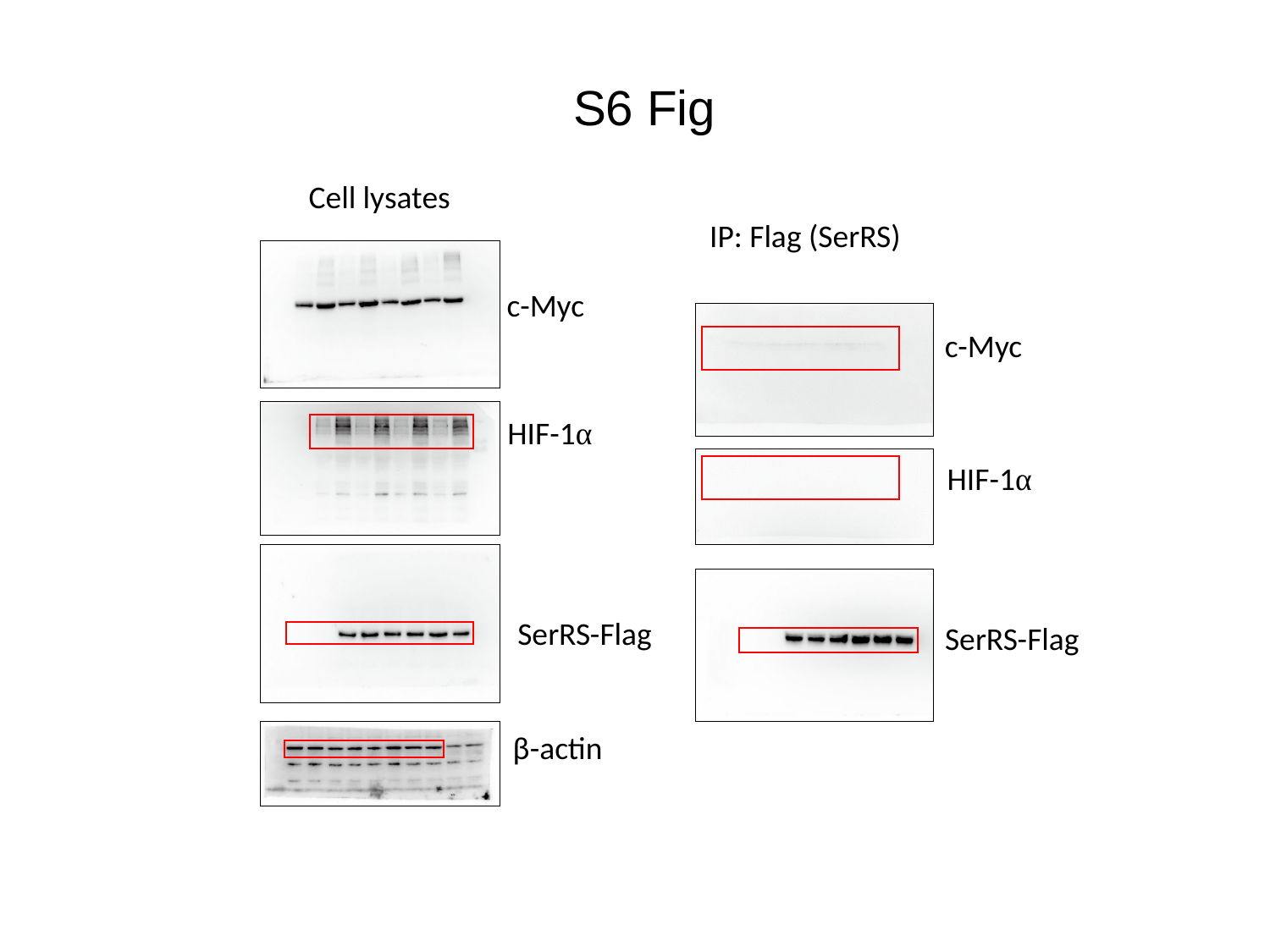

S6 Fig
Cell lysates
IP: Flag (SerRS)
c-Myc
c-Myc
HIF-1α
HIF-1α
SerRS-Flag
SerRS-Flag
β-actin

## Slide 18
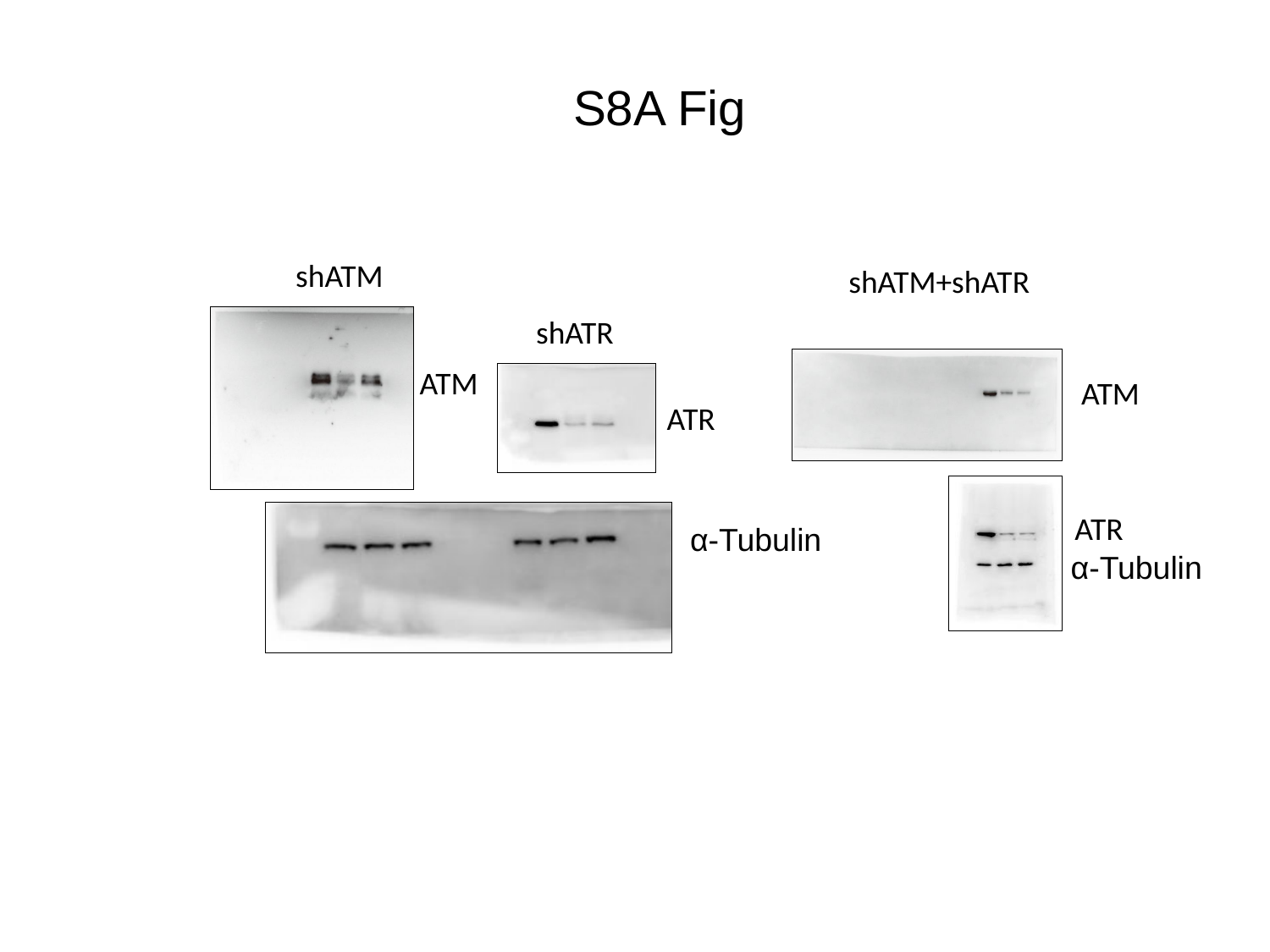

S8A Fig
shATM
shATM+shATR
shATR
ATM
ATM
ATR
ATR
α-Tubulin
α-Tubulin
