# Supplementary material for: Phosphorylation of seryl-tRNA synthetase by ATM/ATR is essential for hypoxia-induced angiogenesis
Source: PLoS Biol. 2020 Dec 22;18(12):e3000991. doi: 10.1371/journal.pbio.3000991 (PMC7755189; doi:10.1371/journal.pbio.3000991)
Supplement: Attachment — Submitted filename: PLOS Biology review.docx [file pbio.3000991.s014.docx]

In this submitted manuscript, Shi et al. performed a series of studies in human cells, zebrafish, and mice, to identify a novel molecular mechanism in hypoxia-induced VEGF in both endothelial cells and cancer cell line. Hypoxia phosphorylates SerRS at Ser101 and Ser241 through ATM/ATR, which diminishes the binding of SerRS in the promoter region of VEGF and attenuates the transcriptional repress effect of SerRS. Furthermore, they found the dissociation of SerRS in the promoter of VEGF is required for HIF1a and c-Myc binding. As the functional readout, SerRS WT or SerRS AA abolishes hypoxia-induced angiogenesis. Overall, the study is nicely executed and the conclusion is convincing. Addressing the following points could further strengthen the manuscript.

1. The involvement of SerRS phosphorylation, HIF1a, c-Myc, and VEGF expression under hypoxia has been well documented. The regulation between SerRS phosphorylation and HIF1a, c-Myc binding is interesting. The mechanistic study could be further strengthened by addressing the relationship and balance between transcriptional repressors and activators in the VEGF promoter. For example, why SerRS dissociation from the VEGF promoter is required for HIF1a and c-Myc binding? Whether the SerRS binding site is near to the HIF1a or c-Myc binding site? The authors showed the SerRS AA form abolished hypoxia-induced c-Myc and HIF1a binding. To further validate this finding, the author should study whether deletion or mutation of SerRS binding site in a reporter system (e.g., VEGF promoter-Luci reporter plasmid) can rescue the SerRS AA decreased c-Myc or HIF-1a binding and gene expression?

2. In HUVECs, is the hypoxia-induced phosphorylation of SerRS also through ATM/ATR? The authors should consider to detect p-ATM in HUVECs under hypoxia (Figure 1G).

3. In Figure 2B, under SerRS knockdown (SerRS-MO), SerRS AA caused an increase in VEGF mRNA expression when compared with SerRS WT. As a de-phosphomimetics form, SerRS AA should cause more transcriptional repression effect than Ser WT, at least should have the comparable VEGF expression level under normoxia condition. The authors need to clarify this discrepancy.

4. In Figure 4A and 4B, the comparisons were made between SerRS WT and SerRS AA under normoxia and hypoxia. Since SerRS DD strongly induced SerRS release from VEGF promoter, a comparison between SerRS WT and SerRS DD is essential to reveal the VEGF regulatory effect of SerRS phosphorylations.

5. Data showed in Figure 1 and Figure 3 showed that SerRS had a low level of phosphorylation and high level of DNA binding under normoxia. If so, SerRS WT and SerRS AA should have comparable DNA binding. However, Figure 3F shows that the DNA binding of SerRS was decreased in SerRS AA transfection when compared with WT. SerRS DD form should be involved in Figure 3F, especially under normoxia condition.

6. As the major evidence of endothelium related angiogenesis, the CD31 or vWF staining is needed in the figure 5.

7. The last subtitle in Results “SerRS AA strongly inhibits tumours angiogenesis in mice”, but the data indicated there was no extra suppression effect of SerRS AA in VEGF expression and angiogenesis when compared with WT. As discussed by the authors, this might be due to the high level of SerRS WT overexpression saturated the phosphorylation capacity of ATM/ATR. But in vitro data showed the decreased VEGF and angiogenesis in AA compared with WT (e.g., Figure 4), even under the much higher level of SerRS overexpression. The authors should future discuss the discrepancy or weaken this subtitle.

8. The western blot in Figure 6E showed the higher expression levels of SerRS in the AA group than the WT group, but the bar graph in Figure 6F presented the opposite trend.

9. The detailed methods about SerRS WT, SerRS AA, or SerRS DD stable transfection in HUVECs are needed.

10. In Figure 1B, the labeling on Y-axis should be “VEGFA induction”.
