# Supplementary material for: Phosphorylation of seryl-tRNA synthetase by ATM/ATR is essential for hypoxia-induced angiogenesis
Source: PLoS Biol. 2020 Dec 22;18(12):e3000991. doi: 10.1371/journal.pbio.3000991 (PMC7755189; doi:10.1371/journal.pbio.3000991)
Supplement: Attachment — Submitted filename: Response to reviewers 2020.08.01.final.docx [file pbio.3000991.s015.docx]

**Point-by-point Response to Reviewers' comments**

First of all, we would like to thank all reviewers for their most helpful input and comments. We believe the paper has been significantly strengthened as a result of addressing them. Despite the challenges imposed by the pandemic and the temporary closure of our labs, we managed to perform many new experiments. These include a complete redone of the MDA-MB-231 xenograft experiment to study the effect of SerRS (and its mutants) not only on angiogenesis but also on tumor growth in mice, as well as monitoring the phosphorylation status of SerRS in the xenografts. The result strongly demonstrates that SerRS is a key negative regulator of angiogenesis and tumor growth *in vivo* and that its natural inactivation by phosphorylation is required for angiogenesis to occur to sustain tumor growth. We also probed for a potential interaction between SerRS and c-Myc/HIF-1 under both normaxia and hypoxia conditions as suggested by reviewer 1. The result ruled out this possibility and strengthened our conclusion that SerRS inhibits c-Myc and HIF through competitive DNA binding. Other new experiments (including a new luciferase assay) and revisions are detailed in the point-by-point responses below. All major changes we made are highlighted in red on the revised manuscript; new or updated figures are indicated by green boxes.

**Reviewers’ comments**

**Rev. 1:**

General Comment:

In this manuscript, the authors proposed a mechanism leading to SerRS inactivation that enables cMyc- and HIF1a-mediated expression of VEGFA in response to hypoxia.

The authors showed that hypoxia activates ATM/ATR, which subsequently phosphorylate SerRS on specific residues (S101 and S241). Phosphorylation of SerRS decreases the its affinity for the VEGFA promoter. Thus, ATM/ATR release transcriptional repression that SerRS has on VEGFA promoter. This affects the ability of SerRS to induce EC proliferation and angiogenesis in vitro and in vivo.

The authors have previously shown that SerRS transcriptionally represses VEGFA by direct DNA binding to its promoter region and recruitment of the histone deacetylase Sirt2 (Ref 19). In addition, they had shown that SerRS counteracts the transcriptional activation activity of c-Myc on VEGFA promoter. Thus, the major novelty in this work is the concept that ATM/ATR regulates SerRS as a transcriptional repressor in response to hypoxia. It is worth noting that a similar claim has been published recently by Song Y et al. Cancers (Basel) 2019 Nov 22;11(12). Song Y et al. reported the same residues being targeted by ATM/ATR kinases, and used similar approaches, including SerRS point mutants. Yet, their focus as a trigger for ATM/ATR activation was UV-mediated DNA double-strand breaks.

These findings are conceptually relevant, the manuscript is wells structured and experimental designs well organized.

For further clarity and confirmation of the proposed working model, authors should address the following major and minor issues:

Major points:

1. One of the major claims related to this work is that ATM/ATR are activated by hypoxia, which in turn negatively regulates SerRS by phosphorylation, leading to activation of VEGF expression. However, in this study, hypoxia was induced by a chemical approach. Glucose oxidase has been reported to induced H2O2, which induces DNA damage. Do authors' hypoxia protocol generates DNA damage? This would be a source of confounding effects related to the method of ATM/ATR activation. Please confirm with gammaH2AX stainings, or alternative methods. Can authors replicate the key results with a hypoxic chamber?

We thank the reviewer for pointing out the potential caveat of the chemical approach we used. In fact, we have attempted to use a hypoxia chamber but unfortunately experienced difficulties in maintaining a hypoxic environment for a prolonged period of time, due to potential leakage of our device. We therefore switched to the two-enzyme approach, which has been well-established to induce hypoxia for cell-based experiments (Raymond P. Baumann et al., *Radiat Res*. 2008, doi:10.1667/RR1431.1). This approach uses a vast excess of catalase (120 U/ml) in addition to glucose oxidase (2 U/ml). Glucose oxidase catalyzes an oxygen-depletion reaction, which does produce H2O2, as pointed out by the reviewer. However, the H2O2 is immediately converted to water by the catalase in the medium. To make sure there was no oxidative stress-induced DNA damage, which can also activate ATR/ATM, we have monitored H2AX phosphorylation (γ-H2AX). As shown in the figure below, in contrast to UV treatment, hypoxia generated by the two-enzyme approach did not appear to induce H2AX phosphorylation. We have now added this explanation on page 7 and the data as S2 Fig in the revised manuscript.

We would like also to point out that we now have succeed in detecting SerRS phosphorylation in tumor xenograft tissues where a hypoxic environment occurs naturally. As shown in the new Fig 6K and 6L (see below), the phosphorylation level of SerRS as detected by specific ATM/ATR substrate (p-S*Q) antibody was significantly reduced in tumors expressing SerRS mutants (SerRS^AA^ or SerRS^DD^) with disrupted ATM/ATR substrate sites (S101 and S241), compared to SerRS^WT^, confirming that ATM/ATR-mediated SerRS phosphorylation indeed occurs under the hypoxic tumor microenvironment.

Fig 6K and 6L.

2. Authors should confirm that inhibition of ATM/ATR leads to reduced VEGF expression under hypoxic conditions (Fig.3H) using siRNA-mediated KD [single and combination] of ATM and ATR. Similarly, authors should confirm results in Fig.1I using the inhibitors.

As suggested, we performed the knockdown experiment. Using shRNA-mediated ATM/ATR knockdown, we show that knockdown of either ATM or ATR inhibits VEGFA induction under hypoxia and it is unclear whether the dual knockdown has a stronger effect, confirming that both ATR and ATM are major players in stimulating VEGFA expression during hypoxia. The new data is shown below and included as S7 Fig in the revised manuscript.

In the original submission, we had already included data showing that SerRS phosphorylation under hypoxia could be blocked by ATM and ATR inhibitors (S1D Fig).

3. In Fig. 2A, it is not clear why overexpression of SerRS WT would lead to a decrease in VEGFA production in non-hypoxic conditions? Does that mean that SerRS is rate limiting? Yet, it still responds with the same magnitude to hypoxic stimulus Fig. 3G. Can authors comment on this observation?

Under normoxia, SerRS is not phosphorylated and therefore is active in binding to *VEGFA* promoter to repress its expression (as shown in Fig 2A). In our previous publications (https://www.nature.com/articles/ncomms1686.pdf and <https://elifesciences.org/articles/02349>), we showed that SerRS counteracts Myc in transcription regulation of VEGFA. Under non-hypoxic conditions, their counteraction reaches an equilibrium. However, overexpression of SerRS^WT^ would push the equilibrium towards SerRS dominance and further decrease VEGFA production.

The WT SerRS (whether it is overexpressed or endogenous) can be inactivated by phosphorylation under hypoxia conditions. This is why cells expressing only endogenous SerRS (Vector control) and cells overexpressing SerRS^WT^ can respond to a similar magnitude to hypoxic stimulus (in term of VEGFA induction) as shown in Fig. 3G.

In order to highlight VEGFA induction, in Fig. 3G we normalized *VEGFA* expression in hypoxia condition to that in normoxia for each group (note the Y axis is Hypoxia/Normoxia). In fact, if we plot the Y-axis as VEGFA mRNA level as shown below, it is clear that overexpression of SerRS^WT^ represses *VEGFA* expression, consistent as that shown in Fig. 2A. We have included this additional plot in Fig. 3G to clarify.

Fig 3G.

4. In Fig.3B/F, it is surprising that the binding of SerRS AA to VEGFA promoter is not greater than that of SerRS WT in normoxic but specially in hypoxic conditions. Could the authors comment on this?

SerRS AA (SerRS S101A/S241A) was designed to block phosphorylation by ATM/ATR, so that it cannot be inactivated under hypoxia. In contract, SerRS WT can be phosphorylated and inactivated under hypoxia. The AA (S101A/S241A) double mutation per se does not affect the VEGFA promoter binding activity of SerRS (Fig. 3A and 3B). Therefore, the binding of SerRS AA to VEGFA promoter is similar to that of SerRS WT in normoxia (Fig. 3F). However, under hypoxia, only SerRS WT (but not SerRS AA) can be phosphorylated by ATM/ATR, which inhibits the VEGFA promoter binding activity of SerRS (Fig. 3F).

Since SerRS AA does prevent HIF1a and Myc from binding to VEGFA promoter (Fig.3F), this suggests that SerRS AA interacts with cMyc and HIF1a but not at the DNA level, thus, sequestering them and preventing their binding to VEGFA promoter. Changes in protein levels and/or post-translational modifications on HIF1a and Myc in response to hypoxia could also influence the output of the competition.

To clarify these different possibilities the authors could:

a. Perform an EMSA to evaluate a possible competition between SerRS WT and SerRS AA together with c-MYC for binding to VEGFA proximal promoter.

We performed the EMSA analysis in our previous work (<https://elifesciences.org/articles/02349>), which have demonstrated the competition between SerRS and c-Myc (with its partner MAX) on binding to VGEFA promoter DNA (see figure below). As the AA (S101A/S241A) double mutation per se does not affect the DNA binding activity of SerRS (as shown by Fig. 3A and 3B), a competition between SerRS^AA^ and c-Myc would be expected as well.

To confirm this point, we have re-performed the ChIP-qPCR analysis, in which expression of SerRS^AA^ prevents c-Myc from binding to the VEGFA promoter with or without hypoxia (new Fig 3F; right).

Fig 3F.

b. Perform a WB for HIF1a and c-Myc/c-Myc phosphorylated under the experimental conditions shown in Figure 3D and E.

c. Attempt to co-IP SerRS isoforms together with HIF1a or Myc.

As suggested by the reviewer, we have performed the WB and Co-IP experiment to test the possibility that SerRS AA interacts with c-Myc and HIF1a and thus sequestering them and preventing them from binding to VEGFA promoter. As shown in the figure below, overexpression of SerRS^WT^ or its mutants does not affect the protein levels of Myc and HIF-1 in the cell. And no interaction with c-Myc/HIF-1 can be detected for SerRS, including SerRS^AA^, thus ruling out the possibility that SerRS^AA^ interacts and sequesters c-Myc and HIF1a to influence their DNA-binding. Although this is a negative result, it does strengthen our conclusion that SerRS inhibits c-Myc and HIF through competitive DNA binding. We have included this new data as S5 Fig in the revised manuscript.

d. Perform the same experiment as in Figure 3F but using the SerRS DD construct to show if absence of SerRS DD binding to DNA would make the cMyc and HIF1a binding as high as with SerRS WT version.

As suggested, we re-performed the ChIP-qPCR experiment with SerRS DD included. As shown the new Fig 3F (above), while the binding of SerRS^WT^ to the VEGFA promoter decreases under hypoxia, the binding of SerRS^AA^ and SerRS^DD^ no longer respond to change in the oxygen level. As the reviewer predicted, no difference was found in HIF-1α binding to the VEGFA promoter between cells expressing SerRS^WT^ and SerRS^DD^. Interestingly, the expression of SerRS^DD^, compared with SerRS^WT^, further stimulates c-Myc binding to the VEGFA promoter. This is likely due to the fact that Myc is constitutively present in the cell, whereas HIF is only stabilized under hypoxia. The Myc binding to the VEGFA promoter can be inhibited by SerRS^WT^, but not SerRS^DD^, whereas no HIF protein is present under normoxia to be inhibited by SerRS. As mentioned, the new Fig 3F is included in the revised manuscript.

5. The interpretation of zebrafish experiments is a bit puzzling and should be further discussed in the text. The rescue of endogenous SerRS KD by both hSerRS WT and hSerRS AA tends to say that VEGFA is actively repressed in zebrafish embryos in cells that do not contribute for normal angiogenesis at these stages. Otherwise, it could be expected that hSerRS AA would abrogate VEGFA production and thus impair angiogenesis. Does this mean that ATR/ATM-mediated regulation of VEGFA production does not apply to zebrafish angiogenesis? Please comment in results/discussion.

The zebrafish were not under hypoxia, in which condition SerRS WT and SerRS AA are functionally equivalent. ATR/ATM are not activated under normoxia. We clarified this point on page 8 in the revised manuscript.

Minor points

General

1. It is not clear from the figure legends or Material and Methods how many biological replicates were performed in WB experiments, for instance Fig.1F-I. If more than 1 experiment was performed, quantifications would be valuable.

At least two biological replicates were performed for those experiments and consistent results were obtained. We have now added the information on biological replicates in the figure legend.

Abstract

2. The following sentence does not accurately describe the results, since results show that hypoxia does not affect the binding efficiency of SerRS to the VEGFA promoter: "Moreover, expression of SerRS S101A/S241A, a phosphorylation-deficient and constitutively active mutant, prevents hypoxia-induced DNA binding, which is necessary to activate c-Myc and HIF-1 for binding to the VEGFA promoter." Please correct.

Actually, our results show that hypoxia DOES affect the binding efficiency of SerRS to the VEGFA promoter. Hypoxia activates ATM and ATR, which phosphorylate SerRS to remove it from VEGFA promoter. Nevertheless, we modified the sentence to make it more clear.

Results

1. It is not clear from the figure legends or Material and Methods how many biological replicates were performed in WB experiments, for instance Fig.1F-I. If more than 1 experiment was performed, quantifications would be valuable.

This comment is addressed above under “General”.

2. For clarity, the authors should describe in the text what "substrate (p-S*Q) antibody" is.

We now clarified the antibody as “ATM/ATR substrate (p-S*Q) antibody”.

3. For clarity, the authors should improve the description of the results in Figure 1E. Specifically, describe (in the text or figure legend) what Ni-NTA is and describe in the text the result for pATM, pRPA.

We clarified this point on the figure and changed “Ni-NTA purified” to “Purified protein”.

4. The title of the section "Phosphorylation inactivates SerRS as a transcriptional repressor in cells and in vivo" does not really match the findings described in this same section. In this section, the authors show that SerRS phosphorylation affects VEGFA expression and affects angiogenesis in vivo but do not show that phosphorylation affects SerRS transcriptional repressor activity. The effect of phosphorylation on transcriptional repressor activity of SerRS is shown only in the next section. Therefore, the title of the section should be rephrased to match more exactly the findings.

We thank the reviewer for pointing this out. We have changed the title of the section to “Phosphorylation inactivates SerRS regulating VEGFA in cells and *in vivo”.*

5. In Fig.6, it is very surprising that no tumor growth curves were measured.

We have now completely redone the MDA-MB-231 xenograft experiment and monitored tumor growth. In addition to monitoring angiogenesis through VEGFA and VE-cadherin levels, we now also measured tumor growth and detected the phosphorylation status of SerRS in the tumor. As shown below, strong inhibitory effect of SerRS^WT^ and SerRS^AA^, but not of SerRS^DD^, was observed on tumor growth. The AA mutation (SerRS^AA^) significantly enhanced the inhibitory effect of SerRS^WT^. In fact, SerRS^AA^-expressing MDA-MB-231 cells barely grew into tumors in mice. The result is now included in the new Fig 6 in the revised manuscript. Please refer to the revised manuscript for more details.

Fig 6A-6C.

6. In Fig.4C/D, data on anti-VEGF antibody. Do the results mean that VEGF has no role in basal conditions? Please comment.

As shown in Fig. 4B, the basal VEGFA level under normoxia is very low (~10 pg/mL/mg total proteins). Therefore, there is little room for anti-VEGFA antibody to inhibit and to show an effect in the tube formation assay.

7. Experiments using matrigel in Fig.4. The description in the text and figure legend is very unclear for readers. It is not obvious which cells were transfected and/or subjected to hypoxia or anti-VEGFA. Authors should make label figure in a more precise way so it becomes obvious that HUVECs in matrigel were not subjected to hypoxia and transfection, but rather only HUVECs that produced the supernatant.

The Matrigel experiment is shown in Fig. 5 (not Fig. 4) and was performed with mouse 3B11 endothelial cells (not HUVEC cells). There was no hypoxia or anti-VEGFA treatment. The reviewer might have confused this experiment (Fig. 5) with the Fig. 4 experiment and its figure legend.

8. Figure 5 B, 6A and 6C - stainings are not easy to see - consider changing colors to facilitate visualization (e.g. - grayscale inverted + green). In addition, the resolution of the images is very low. In addition, the resolution of input images and file compression should be revised.

We now have replaced Fig 5B with higher resolution images and replaced the entire Fig 6 with new data and better images (Fig 6G and 6I) in the revised manuscript.

Fig 5B.

Fig 6G and 6I.

Discussion

1. The Figure S7 lacks details (efficiency of SerRS knockdown, how the experiment was performed (timing of analysis after shRNA transfection in the figure legend). Also, the hypothesis that SerRS transcriptionally regulates IL8 expression (as a direct target) and that it can antagonize ROS/NF-kB/IL8 hypoxia response is, at this stage, highly speculative. Therefore, given the lack of solid evidence for such conclusion, I suggest the authors the removal this part from the working model in Figure 7.

We thank the reviewer for this comment. We have now expanded the figure (new S10 Fig) to include: 1) WB result to demonstrate SerRS knockdown efficiency; and 2) a list of 215 angiogenesis related genes included in the analysis to provide more details of this experiment. We also clarified in the revised manuscript (page 16) that IL-8 may not be a direct target gene of SerRS. We believe including this *ROS/NF-kB/IL-8* pathway, although speculative, can help provide a broader understanding of SerRS in angiogenesis regulation.

Figure legends

1. Figure 1E - Ni-NTA should be described in the figure legend.

We revised Fig 1E by changing “Ni-NTA purified” to “Purified protein” to provide clarity.

2. Figure 3B - In the figure, fold change is shown. In the legend, it should be specified how the fold change is represented.

The figure legend is revised as suggested.

*Materials and Methods*

*1. It would be helpful to provide more accurate information on the following: "Hypoxia medium was prepared by diluting glucose oxidase and catalase at a constant 1:10 ratio in cell culture medium". Could you please specify the final concentration in solution of these enzymes?*

The concentration of the two enzymes are as follows: glucose oxidase (2 U/ml) and catalase (120 U/ml). This information has been added to the Material and Methods.

*2. Anti-HIF-1β (ARNT, #3414) antibody was not used in the study. It should be removed from the the Materials and methods section.*

Removed as suggested.

*Typographical errors*

We thank the reviewer for pointing out those errors.

*The MS contains several typos and imprecisions that should be corrected. Just to highlight a very obvious one, the title "Phosphorylation of a tRNA synthetase by ATM/ATR essential for hypoxia-induced angiogenesis" is missing a verb.*

A verb is added.

*A few other below:*

*1. All Western Blot figures - "KD" refers to kilodaltons, should be spelled "kDa"*

Revised as suggested.

*2. Figure 1 B - y axis - "VEGFA indiction" should be spelled "VEGFA induction"*

Revised as suggested.

*3. Figure 1 C - "H. sapiesn" should be spelled "H. sapiens"*

Revised as suggested.

*4. Figure 1 E - "Nulear extracts" should be spelled "Nuclear extracts"*

Revised as suggested.

*5. Abstract: "Moreover, expression of SerRSS101A/S241, a phosphorylation-deficient and constitutively active mutant…" should be spelled "Moreover, expression of SerRSS101A/S241A, a phosphorylation-deficient and constitutively active mutant…"*

Revised as suggested.

*6. Results: "*

*We further confirmed the expression of SerRS and the molecular markers for vasculature in the tumours xenografts by Western bolt analysis"*

*should be spelled "*

*We further confirmed the expression of SerRS and the molecular markers for vasculature in the tumours xenografts by Western blot analysis"*

*Revised as tumor xenografts.*

*7. Discussion: "*

*Tumours cells can adapt to the hypoxic environment through orchestrating the expression of a large number of genes that functions in apoptosis, cell survival, energy metabolism, and angiogenesis"*

*should be spelled "*

*Tumour cells can adapt to the hypoxic environment through orchestrating the expression of a large number of genes that function in apoptosis, cell survival, energy metabolism, and angiogenesis"*

Revised as suggested.

*8. Discussion: "or example, K-RAS and hypoxia tension can synergistically increase the level of GTP-bound Rho" should be spelled: "For example, K-RAS and hypoxia tension can synergistically increase the level of GTP-bound Rho"*

Revised as suggested.

***Rev. 2:***

In this submitted manuscript, Shi et al. performed a series of studies in human cells, zebrafish, and mice, to identify a novel molecular mechanism in hypoxia-induced VEGF in both endothelial cells and cancer cell line. Hypoxia phosphorylates SerRS at Ser101 and Ser241 through ATM/ATR, which diminishes the binding of SerRS in the promoter region of VEGF and attenuates the transcriptional repress effect of SerRS. Furthermore, they found the dissociation of SerRS in the promoter of VEGF is required for HIF1a and c-Myc binding. As the functional readout, SerRS WT or SerRS AA abolishes hypoxia-induced angiogenesis. Overall, the study is nicely executed and the conclusion is convincing. Addressing the following points could further strengthen the manuscript.

1. The involvement of SerRS phosphorylation, HIF1a, c-Myc, and VEGF expression under hypoxia has been well documented. The regulation between SerRS phosphorylation and HIF1a, c-Myc binding is interesting. The mechanistic study could be further strengthened by addressing the relationship and balance between transcriptional repressors and activators in the VEGF promoter. For example, why SerRS dissociation from the VEGF promoter is required for HIF1a and c-Myc binding? Whether the SerRS binding site is near to the HIF1a or c-Myc binding site? The authors showed the SerRS AA form abolished hypoxia-induced c-Myc and HIF1a binding. To further validate this finding, the author should study whether deletion or mutation of SerRS binding site in a reporter system (e.g., VEGF promoter-Luci reporter plasmid) can rescue the SerRS AA decreased c-Myc or HIF-1a binding and gene expression?

As suggested, we have performed a luciferase assay by putting the VEGFA promoter (-1005 to +379) in front of a luciferase reporter gene to test the transcriptional activity of SerRS^AA^. Our previous work has identified the SerRS binding site (SBS) on the VEGFA promotor to be a 27-bp region (-62 to -36) that overlaps with the E-box binding site of Myc (-49 to -44) (<https://elifesciences.org/articles/02349>). The HIF-binding site on the VEGFA promotor is located upstream (-975 to -968). As shown in the new figure (S6 Fig) below, the expression of SerRS^AA^ inhibits the hypoxia induced luciferase activity and this inhibition is dependent on SerRS-DNA binding, as removal of SBS abolishes the inhibitory effect of SerRS^AA^.

Interestingly, under normoxia, with or without the expression of SerRS^AA^, removal of SBS leads to an increased reporter expression, which is consistent with the loss of transcription inhibition by endogenous SerRS due to the SBS deletion.

These data further supported that the binding of phosphor-deficient SerRS^AA^ on *VEGFA* promoter inhibited HIF-1/c-Myc-induced VEGFA expression under hypoxia, highlighting that hypoxia-induced phosphorylation and release of SerRS from *VEGFA* promoter is required for efficient induction of *VEGFA* by other positive regulator, such as HIF-1 and c-Myc. Although, unlike c-Myc, the HIF-1 binding site does not directly overlap with the SerRS binding site, we speculate that the binding of SerRS and its partner SIRT2, a histone deacetylase, would cause chromatin modification/condensation in the neighbor regions and therefore indirectly affect the recruitment/binding of other transcriptional factors, such as HIF-1.

We have included this new data in the revised manuscript as S6 Fig.

*2. In HUVECs, is the hypoxia-induced phosphorylation of SerRS also through ATM/ATR? The authors should consider to detect p-ATM in HUVECs under hypoxia (Figure 1G).*

We have now added the WB analysis on p-ATM in the revised Fig 1G.

*3. In Figure 2B, under SerRS knockdown (SerRS-MO), SerRS AA caused an increase in VEGF mRNA expression when compared with SerRS WT. As a de-phosphomimetics form, SerRS AA should cause more transcriptional repression effect than Ser WT, at least should have the comparable VEGF expression level under normoxia condition. The authors need to clarify this discrepancy.*

We totally agree that “*SerRS AA should cause more transcriptional repression effect than SerRS WT, at least should have the comparable VEGF expression level under normoxia condition.”* The lower activity of SerRS^AA^ in this experiment was due to the lower expression level of SerRS^AA^ than SerRS^WT^. However, because the expression level of SerRS^AA^ and SerRS^DD^ was similar, yet only SerRS^AA^ but SerRS^DD^ can suppress VEGFA expression, we can conclude that the phosphorylation event at S101 and S241 sites is responsible for inhibiting the transcriptional repressor activity of SerRS on VEGFA *in vivo*. We have clarified this discrepancy in the revised manuscript on page 8 - “The partial activity of SerRS^AA^ is likely due to the lower expression level of SerRS^AA^ compared with SerRS^WT^ (Fig 2B).”

*4. In Figure 4A and 4B, the comparisons were made between SerRS WT and SerRS AA under normoxia and hypoxia. Since SerRS DD strongly induced SerRS release from VEGF promoter, a comparison between SerRS WT and SerRS DD is essential to reveal the VEGF regulatory effect of SerRS phosphorylations.*

We have now added the statistical analysis between WT and DD in the revised Fig 4A and 4B.

Fig 4A and 4B.

*5. Data showed in Figure 1 and Figure 3 showed that SerRS had a low level of phosphorylation and high level of DNA binding under normoxia. If so, SerRS WT and SerRS AA should have comparable DNA binding. However, Figure 3F shows that the DNA binding of SerRS was decreased in SerRS AA transfection when compared with WT. SerRS DD form should be involved in Figure 3F, especially under normoxia condition.*

As suggested, we have repeated the ChIP-qPCR experiment with SerRS DD included (new Fig 3F; see below). Our new result confirmed that SerRS WT and SerRS AA have comparable DNA binding under normaxia, as indicated by the reviewer. The new Fig 3F is included in the revised manuscript.

Fig 3F.

*6. As the major evidence of endothelium related angiogenesis, the CD31 or vWF staining is needed in the figure 5.*

CD31 staining result is included in S8B and S8C Fig.

*7. The last subtitle in Results “SerRS AA strongly inhibits tumours angiogenesis in mice”, but the data indicated there was no extra suppression effect of SerRS AA in VEGF expression and angiogenesis when compared with WT. As discussed by the authors, this might be due to the high level of SerRS WT overexpression saturated the phosphorylation capacity of ATM/ATR. But in vitro data showed the decreased VEGF and angiogenesis in AA compared with WT (e.g., Figure 4), even under the much higher level of SerRS overexpression. The authors should future discuss the discrepancy or weaken this subtitle.*

We thank the reviewer for emphasizing this important point. To clarify, we have now completely redone the *in vivo* experiment and obtained new results that are consistent with an enhanced angiogenesis inhibition activity of SerRS-AA, compared with SerRS-WT (new Fig 6D-6J), as we would expect from the *in vitro* data. In addition to monitoring angiogenesis through VEGFA and VE-cadherin levels, we now also measured tumor growth. The phosphor-deficient AA mutation (SerRS^AA^) significantly enhanced the tumor inhibitory effect of SerRS^WT^. In fact, SerRS^AA^-expressing MDA-MB-231 cells barely grew into tumors in mice (new Fig 6A-6C). Moreover, we now also succeed in detecting SerRS phosphorylation in the tumor tissues. The specific phosphorylation level was significantly reduced in tumors expressing either SerRS^AA^ or SerRS^DD^, compared to SerRS^WT^ (new Fig 6K and 6L), confirming that SerRS phosphorylation at S101 and/or S241 indeed occurs under hypoxic tumor microenvironment to inactivate the anti-angiogenic function of SerRS to allow sustained tumor growth. These new data are presented in the new Fig 6 and new S9 Fig.

*8. The western blot in Figure 6E showed the higher expression levels of SerRS in the AA group than the WT group, but the bar graph in Figure 6F presented the opposite trend.*

As stated above, Fig 6 has now been completely replaced with data from a new experiment, and the expression level of SerRS proteins (WT, AA, DD) have been quantified in S9 Fig, which indicates equal expression levels among different SerRS proteins (see above).

*9. The detailed methods about SerRS WT, SerRS AA, or SerRS DD stable transfection in HUVECs are needed.*

The information has now been added to Materials and Methods on page 18.

*10. In Figure 1B, the labeling on Y-axis should be “VEGFA induction”.*

The mistake has been corrected.

***Rev. 3:***

In this study the authors analyze the mechanism of hypoxia induced VEGFA expression, by elucidating the transcriptional repressor role of SerRS in normoxic conditions. By combining analysis in human cells, zebrafish and mice, they show that SerRS phosphorylation through ATM and ATR is induced by hypoxia and reduces SerRS DNA binding.

The study is generally well done, experimentally sound and through the comparison of the different models does validate most of the claims by more than one way. Therefore it does not require much additional experimental work for publishing. It is original enough and of importance to researchers in its field or at least to a subset of this audience. However, I doubt it to be of interest to a more general audience, as would be required for publication in Plos biology in its current state. To broaden the impact, the authors should analyze the consequences of SerRS phosphorylation for other c-Myc and Hif-1 targets, ideally even establishing differences between hypoxia dependent and independent targets (at least of c-Myc).

We thank the reviewer for the positive comments on our work and the insightful suggestion to understand more broadly the consequences of SerRS phosphorylation for other c-Myc and HIF-1 targets. In fact, encouraged by the extremely strong inhibition effect of SerRS-AA on tumor growth (much stronger than that of SerRS-WT) (new Fig 6), we are currently investigating this question in the context of tumorigenesis. Given that both c-Myc and HIF-1 are master regulators that drive the oncogenic process, it is conceivable that SerRS phosphorylation has a broad impact on c-Myc and HIF-1 and their target genes beyond VEGFA. However, the current paper is focused on angiogenesis and therefore we respectfully argue that this question, although important, is out of scope of the manuscript.

Minor comments:

The manuscript is well organized and written clearly to allow for understanding of non-specialist. However, throughout the manuscript there is a large number of grammatical and spelling errors, which need to be corrected.

We thank the reviewer for pointing out this issue and have corrected the errors.

Also there seems to be insecurity in terms of proper nomenclature. As the nomenclature rules are different for mouse, human and fish, the authors do need to check each gene/protein and abbreviation for species correct nomenclature.

We have edited the manuscript with correct nomenclatures.

The last sentence of the abstract is pure speculation and meaningless, especially dealing with intracellular transcription factors, which make poor therapeutical targets, therefore the authors should focus on a more strong and study related finish of the abstract.

As suggested, we have rewritten the last sentence of manuscript. We thank the reviewer for this comment.
