# Supplementary material for: Phosphorylation of seryl-tRNA synthetase by ATM/ATR is essential for hypoxia-induced angiogenesis
Source: PLoS Biol. 2020 Dec 22;18(12):e3000991. doi: 10.1371/journal.pbio.3000991 (PMC7755189; doi:10.1371/journal.pbio.3000991)
Supplement: Attachment — Submitted filename: Response to reviewers 2020.11.1.docx [file pbio.3000991.s016.docx]

**Point-by-point Response to Reviewers' comments**

Rev. 1:

The reviewer acknowledges the substantial efforts that were made by the authors to answer all queries. These efforts are even more commendable given the ongoing pandemic restrictions. Authors have answer most of the major and minor concerns and they have clarified many of the initial critics. Therefore, the reviewer supports the publication of this report in PloS Biology. However, there are still some concerns and improvements in the current MS format (related to the first review and not requiring experimental work) that need to be addressed before publication.

Major Point 1- Authors should provide quantification of WB for new Fig.S2.

Fig S2 has been updated in the revised manuscript to provide quantification of the WB.

Major Point 4- new Fig.3F and old Fig.3F differ significantly in the data for SerRS-AA. When looking at the error bars, one would guess that authors exclude old data points. Does this graph only show data for the new experiment? What is the rational to exclude the previous (old) data?

We indeed excluded the old data to make the comparison more accurate. The old Fig.3F only contained SerRS^WT^ and SerRS^AA^ groups, and reviewers suggested us to include SerRS^DD^ in the experiment. We performed the new experiment with all groups (SerRS^WT^, SerRS^AA^, SerRS^DD^) included and with biological triplicates. The result on SerRS^WT^ and SerRS^AA^ was consistent with the old Fig. 3F. In both the new and old experiments, expressing SerRS^AA^ abolished hypoxia-induced binding of Myc and HIF-1 to the VEGFA promotor.

Major Point 5- authors should provide a reference for the following statement "…because the zebrafish are not under hypoxia;".

Moreover, as authors said, zebrafish are not under hypoxia, and thus, ATM/ATR will not be active to phosphorylate SerRS. From these experiments the major conclusion is that SerRS is a constitutive repressor of VEGF expression in zebrafish embryos, unrelated to ATM/ATR activity, as published in <https://elifesciences.org/articles/02349>.

Overall, the zebrafish data does not bring major novelties to the biology of ATM/ATR regulation of SerRS, and the connection between Fig.1 and Fig.2 is non-existent. Thus, zebrafish data should be included as supplementary data. Moreover, the conclusion that ATM/ATR is not involved in SerRS regulation in zebrafish should be made even clearer in the MS.

As suggested, we moved most zebrafish data into to a new supplementary Figure (S3 Fig) in the revised manuscript. The zebrafish used in our experiment were maintained under a normal oxygen level, and therefore was not under hypoxia.

Minor Point 1- The authors have addressed the major concern on the inaccuracy of the sentence. Yet, the new sentence, "Moreover, expression of SerRSS101A/S241A, a phosphorylation-deficient and constitutively active mutant, prevents hypoxia-induced c-Myc and HIF-1 binding to the VEGFA promoter, which is necessary for the transcription factors to activate gene expression.", is still somehow difficult to read.

The reviewer suggests the following:

"Moreover, expression of SerRSS101A/S241A, a phosphorylation-deficient and constitutively active mutant, prevents the hypoxia-mediated binding of c-Myc and HIF-1 to the VEGFA promoter, two transcription factors that activate VEGFA expression."

As suggested, we further revised this sentence to improve clarity.

"Moreover, expression of SerRS^S101A/S241A^, a phosphorylation-deficient and constitutively active mutant, prevents hypoxia-induced binding of c-Myc and HIF-1 to the *VEGFA* promoter, and activation of VEGFA expression."

Minor Point 5 - These new experiments are puzzling. Authors should clarify why SerRS WT overexpression leads to a significant decrease in tumour growth and VEGFA production in tumors. This is incompatible with the model proposed and with experiments showing that overexpression of SerRS WT does not affect levels of VEGFA expression in hypoxic conditions relative to control cells (Fig3G, Fig4A/B). As authors wrote in reply to major point 3 "The WT SerRS (whether it is overexpressed or endogenous) can be inactivated by phosphorylation under hypoxia conditions. This is why cells expressing only endogenous SerRS (Vector control) and cells overexpressing SerRSWT can respond to a similar magnitude to hypoxic stimulus (in term of VEGFA induction) as shown in Fig. 3G." Authors should clarify the discrepancy.

As suggested, we added a paragraph to clarify.

"Consistent with the result from the matrigel plug angiogenesis assay (Fig. 5), SerRS^WT^ exhibited partial, but nevertheless strong, activity in inhibiting tumor angiogenesis and tumor growth (Fig 6). These *in vivo* observations contrast to the more complete inactivation of SerRS^WT^ by hypoxia in cell-based systems (Fig 3G, Fig 4A and 4B). Possibly, the hypoxic environment inside a matrigel plug or a tumor is more heterogenous than in cultured cells, rendering more SerRS^WT^ to remain unphosphorylated and able to inhibit VEGFA expression."

Minor Point 7- Authors did not interpret correctly the reviewer statement.

Fig.4 does use Matrigel for the tube formation assay, and the experiment uses HUVECs, as described in figure legend and material and methods. Please re-interpret the original comment and adapt accordingly. Authors should make clearer that only conditional media, from the defined conditions, was used, instead of using different cell lines assay with normoxia and hypoxia conditions directly on the tube formation assay. The reviewer suggests that a cartoon describing the experimental setup could guide the readers for this important difference.

As suggested, we added a cartoon in Fig. 4 to illustrate the experimental setup.

Rev. 2:

The authors have done an outstanding revision and the manuscript has been significantly strengthened. Since the authors addressed all my concerns in the revised manuscript, I suggest an acceptance of the manuscript.

We thank both reviewers for their most helpful input and suggestions to improve our manuscript.
